# *Msx1* haploinsufficiency modifies the *Pax9*-deficient cardiovascular phenotype

**DOI:** 10.1186/s12861-021-00245-5

**Published:** 2021-10-06

**Authors:** Ramada R. Khasawneh, Ralf Kist, Rachel Queen, Rafiqul Hussain, Jonathan Coxhead, Jürgen E. Schneider, Timothy J. Mohun, Stéphane Zaffran, Heiko Peters, Helen M. Phillips, Simon D. Bamforth

**Affiliations:** 1 Newcastle University Biosciences Institute, Centre for Life, Newcastle, NE1 3BZ UK; 2 School of Dental Sciences, Newcastle University, Newcastle, NE2 4BW UK; 3 Bioinformatics Support Unit, Newcastle University, Newcastle, NE1 3BZ UK; 4 Genomics Core Facility, Newcastle University, Newcastle, NE1 3BZ UK; 5 Biomedical Imaging, University of Leeds, Leeds, LS2 9JT UK; 6 The Francis Crick Institute, London, NW1 1AT UK; 7 INSERM, Marseille Medical Genetics, U1251, Aix Marseille University, Marseille, France; 8 Present Address: Department of Basic Medical Sciences, Faculty of Medicine, Yarmouk University, Irbid, Jordan

**Keywords:** Cardiovascular development, Pharyngeal endoderm, Pax9, Msx1, Neural crest

## Abstract

**Background:**

Successful embryogenesis relies on the coordinated interaction between genes and tissues. The transcription factors *Pax9* and *Msx1* genetically interact during mouse craniofacial morphogenesis, and mice deficient for either gene display abnormal tooth and palate development. *Pax9* is expressed specifically in the pharyngeal endoderm at mid-embryogenesis, and mice deficient for *Pax9* on a C57Bl/6 genetic background also have cardiovascular defects affecting the outflow tract and aortic arch arteries giving double-outlet right ventricle, absent common carotid arteries and interruption of the aortic arch.

**Results:**

In this study we have investigated both the effect of a different genetic background and *Msx1* haploinsufficiency on the presentation of the *Pax9*-deficient cardiovascular phenotype. Compared to mice on a C57Bl/6 background, congenic CD1-*Pax9*^*–/–*^ mice displayed a significantly reduced incidence of outflow tract defects but aortic arch defects were unchanged. *Pax9*^*–/–*^ mice with *Msx1* haploinsufficiency, however, have a reduced incidence of interrupted aortic arch, but more cases with cervical origins of the right subclavian artery and aortic arch, than seen in *Pax9*^*–/–*^ mice. This alteration in arch artery defects was accompanied by a rescue in third pharyngeal arch neural crest cell migration and smooth muscle cell coverage of the third pharyngeal arch arteries. Although this change in phenotype could theoretically be compatible with post-natal survival, using tissue-specific inactivation of *Pax9* to maintain correct palate development whilst inducing the cardiovascular defects was unable to prevent postnatal death in the mutant mice. Hyoid bone and thyroid cartilage formation were abnormal in *Pax9*^*–/–*^ mice.

**Conclusions:**

*Msx1* haploinsufficiency mitigates the arch artery defects in *Pax9*^*–/–*^ mice, potentially by maintaining the survival of the 3rd arch artery through unimpaired migration of neural crest cells to the third pharyngeal arches. With the neural crest cell derived hyoid bone and thyroid cartilage also being defective in *Pax9*^*–/–*^ mice, we speculate that the pharyngeal endoderm is a key signalling centre that impacts on neural crest cell behaviour highlighting the ability of cells in different tissues to act synergistically or antagonistically during embryo development.

**Supplementary Information:**

The online version contains supplementary material available at 10.1186/s12861-021-00245-5.

## Background

Morphogenetic processes rely on tightly regulated gene expression patterns and interactions at critical time points during development. Certain genes may also be utilised at different time points and in different tissues for formation of distinct structures [1]. For example, the transcription factor *Pax9* is required for palate, tooth, taste papillae, thymus, skeletal and cardiovascular development [2–5]. Pax9 is specifically expressed in the pharyngeal endoderm at mid-embryogenesis with wider expression in the craniofacial region and skeletal precursors later in development [6, 7]. Mice lacking *Pax9* die in the neonatal period with cleft palate, tooth agenesis [4] and cardiovascular defects which are the most likely cause of death [2, 3]. Cleft palate is among the most common human birth defects [8], and congenital cardiac anomalies are one of the most frequent disorders associated with cleft lip and palate [9]. Mouse mutants with complete cleft palate die within 24 h of birth without having fed, most likely due to a combination of factors such as the inability to suckle and respiratory distress [10].

Shared specific phenotypes in mutant mouse models can be indicators of a genetic interaction between genes (epistasis) suggesting they are involved in the same gene regulatory network [11]. Pax9 and Msx1, for example, are co-expressed during craniofacial development, and mice deficient for *Msx1* show similar phenotypes to *Pax9*-deficient mice by exhibiting cleft palate and abnormalities of craniofacial and tooth development [4, 12]. Protein–protein interactions occur between Pax9 and Msx1 in vitro [13] and Pax9 and Msx1 interact synergistically in vivo throughout lower incisor development and affect multiple signalling pathways that influence incisor size and asymmetry [14]. Whereas cleft lip formation is incompletely penetrant in *Pax9;Msx1* double homozygous mutants, lower incisors are missing in *Pax9;Msx1* double heterozygotes indicating variable gene dosage requirements in different tissues [14, 15]. Pax9 is able to directly regulate Msx1 expression and interact with Msx1 at the protein level to enhance its ability to transactivate Bmp4 expression during tooth development [16].

*Pax9*-deficient mice have cardiovascular defects affecting the outflow tract and aortic arch arteries, with double outlet right ventricle (DORV), interrupted aortic arch type B (IAA-B), aberrant right subclavian artery (A-RSA) and absent common carotid arteries observed at a high penetrance [3]. These defects are thought to derive from altered signalling processes originating from the pharyngeal endoderm, from a yet to be identified mechanism, but could potentially involve a pathway linking to neural crest cell (NCC) migration or differentiation [3]. A pathway that shares *Pax9* and *Msx1* could be a possibility as the two proteins are known to interact and *Msx1* is expressed in migrating NCC in the pharyngeal arches at E9.5, and these cells differentiate and contribute to the smooth muscle cells (SMC) coating the great arteries [17, 18]. Although *Msx1*-null mice have normal cardiovascular development, *Msx1;Msx2* double homozygous mutant mice die in utero with neural crest, outflow tract and atrioventricular valve defects [19, 20]. Mutation of either *PAX9* or *MSX1* has been implicated in human congenital heart defects [21–26].

Genetic modifiers in different strain backgrounds of mouse models of disease play an important role in phenotype presentation [27], including cardiovascular defects [28, 29]. For example, *Msx1*-deficient mice die more rapidly on a C57Bl/6 background compared to a mixed genetic background [30] and *Pax9;Msx1* homozygous mutants present with a cleft lip on a mixed background, but this is completely suppressed on a CD1 background [14].

In this study we had two objectives: to investigate the *Pax9*-deficient cardiovascular phenotype on a different genetic background to C57Bl/6, and to identify if there was a genetic interaction between *Pax9* and *Msx1* in cardiovascular development.

## Results

**Fig. 1:**
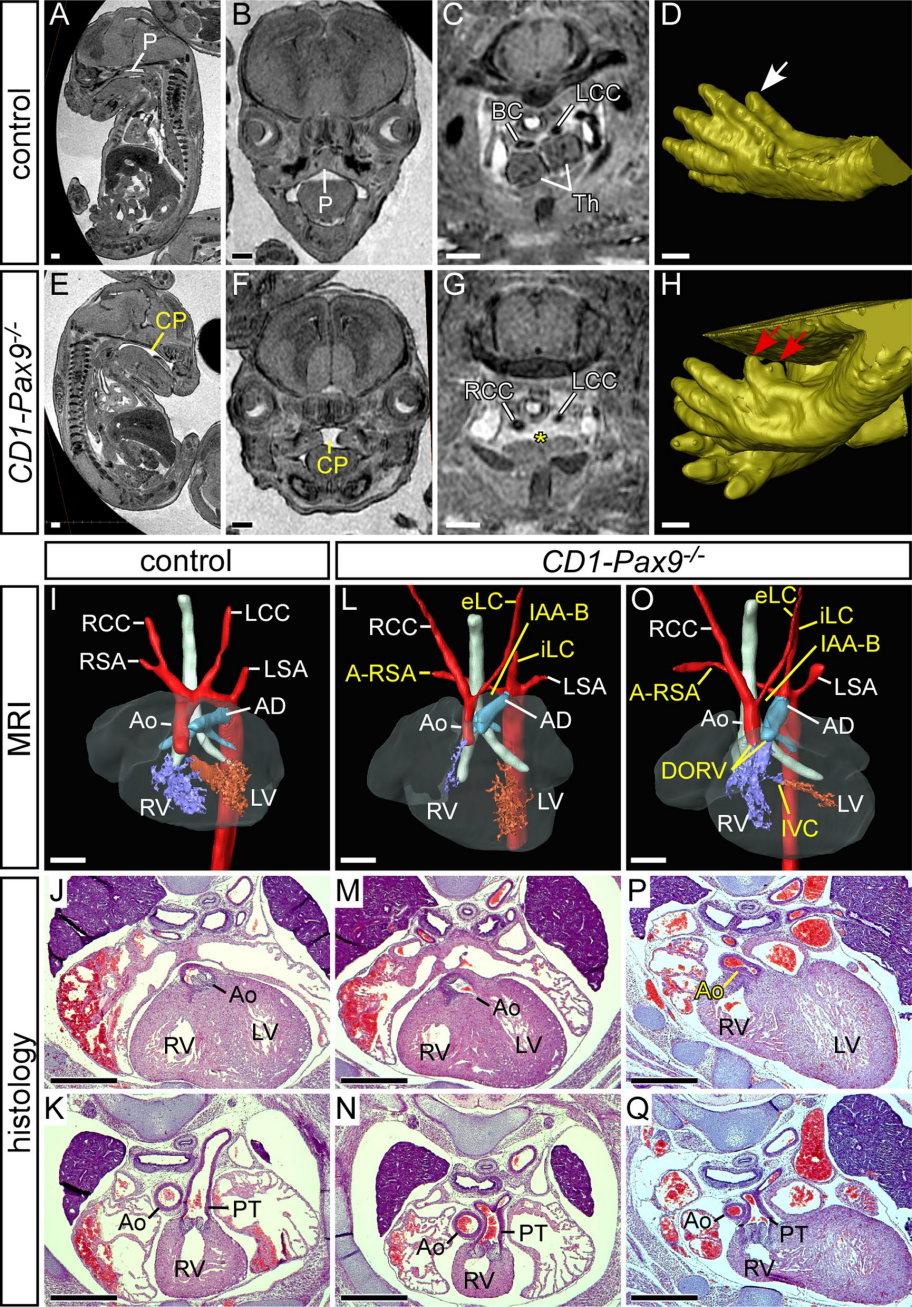
Defects in *Pax9*^*–/–*^ embryos on a congenic CD1 background. Embryos at E15.5 were imaged by MRI (**A**–**H**, **I**, **L**, **O**) and histology (**J**, **K**, **M**, **N**, **P**, **Q**). **A**–**H** Extracardiac defects in CD1-*Pax9*^*–/–*^ embryos. A normal palate (**A**, **B**), thymus (**C**) and hind limb digit (white arrow; **D**) are seen in control embryos. CD1-*Pax9*^*–/–*^ embryos display cleft palate (**E**, **F**), absent thymus (asterisk; **G**) and pre-axial digit duplication (red arrows; **H**). (**I**–**Q**) Cardiovascular defects in CD1-*Pax9*^*–/–*^ embryos. Control embryos have normal heart and aortic arch artery development (**I**–**K**). CD1-*Pax9*^*–/–*^ embryos have arch artery defects such as interrupted aortic arch type B (IAA-B), aberrant right subclavian artery (A-RSA), and absent common carotid arteries resulting in the internal and external left carotid arteries (iLC, eLC) arising directly from the aorta and dorsal aorta respectively (**L**, **O**). In the majority of CD1-*Pax9*^*–/–*^ embryos the outflow tract was unaffected (**L**–**N**) although double outlet right ventricle (DORV) with interventricular communication (IVC) was infrequently observed (**O**–**Q**). AD, arterial duct; Ao, aorta; BC, brachiocephalic artery; CP, cleft palate; LCC/RCC, left/right common carotid artery; LSA/RSA, left/right subclavian artery; LV/RV, left/right ventricle; P, palate; PT, pulmonary trunk; Th, thymus. Scale bars: 500 µm

**Fig. 2:**
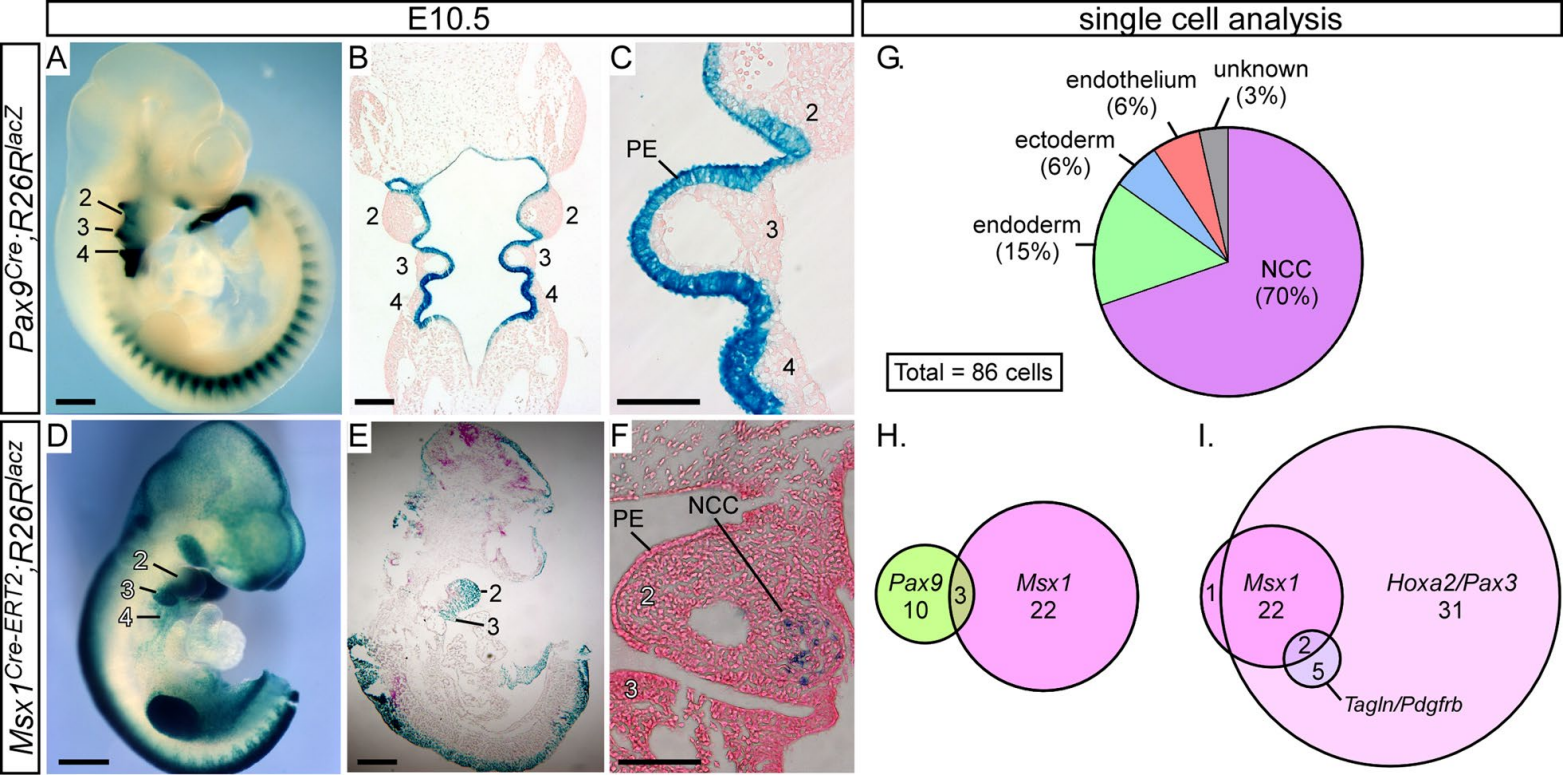
*Pax9* and *Msx1* have non-overlapping expression patterns in the pharyngeal arches. **A**–**F** *Pax9*^*Cre*^ and *Msx1*^*Cre−ERT2*^ mice were mated with *R26R*^*lacZ*^ reporter mice and embryos were stained with X-Gal at E10.5. **A**–**C** *Pax9*^*Cre*^*;R26R*^*lacZ*^ embryo showing *Pax9* expression in the pharyngeal endoderm (PE) in the whole embryo (**A**) and in coronal sections (**B**, **C**). **D**–**F** *Msx1*^*Cre−ERT2*^*;R26R*^*lacZ*^ embryo showing expression in presumed neural crest cells (NCC) in the pharyngeal mesenchyme in the whole embryo (**D**) and in sagittal (**E**) and coronal (**F**) sections. Pharyngeal arches are numbered. Scale bars: 500 µm in **A**, **B**, **D**, **E**, 50 µm in **C**, **F**. **G**–**I** Single cell transcriptomic data from E9.5 caudal pharyngeal arches was analysed for expression of marker genes to identify specific cell types (**G**). *Pax9* and *Msx1* expression was present in only three cells (**H**) and the majority of *Msx1* cells expressed the NCC markers *Hoxa2* and *Pax3* (**I**). A subset of cells also expressed smooth muscle cell markers *Tagln* and *Pdgfrb*

To investigate if the fully penetrant cardiovascular defects seen in mice on a congenic C57Bl/6 genetic background (B6-*Pax9*) were recapitulated on a different genetic background we examined *Pax9*-deficient mice (hereafter referred to as *Pax9*^*–/–*^) which had been backcrossed in excess of 20 generations on an outbred CD1 genetic background (CD1-*Pax9*). These congenic CD1-*Pax9*^+*/–*^ mice were subsequently intercrossed to produce CD1-*Pax9*^*–/–*^ embryos at E15.5 for analysis by MRI, µCT and histology (n = 25). A further 22 neonates were collected on the day of birth and analysed for aortic arch artery defects by dissection and direct visualisation. From a subset of these neonates (n = 12), the heart was removed and further examined for outflow tract and intracardiac defects by histology (Table 1). This analysis revealed that all CD1-*Pax9*^*–/–*^ embryos and neonates presented with a cleft palate, a severely hypoplastic thymus absent from the normal position, and a pre-axial digit duplication (Fig. 1A-H) as previously reported [4]. Surprisingly, CD1*-Pax9*^*–/–*^ mice had a significantly lower incidence of DORV compared to our published data for B6-*Pax9*^*–/–*^ neonates and embryos [3] (n = 24; 16% vs. 79%, *p* < 0.001; Fig. 2E; Table 1), although a very similar incidence of VSD and arch artery defects (IAA and A-RSA) was observed (Figs. 1I–Q; 2F, G; Table 1). Bicuspid aortic valve, however, was not observed. These data demonstrate that a change in genetic background affects the penetrance of the outflow tract defects in CD1*-Pax9*^*–/–*^ mice, although the incidence of arch artery defects was consistent.

**Table 1:** Cardiovascular defects in *Pax9* and *Pax9;Msx1* mutant E15.5 embryos and neonates

Genetic background—genotype	n	VSD	DORV + IVC	cAo	IAA ± A-RSA	A-RSA (w/o IAA)	Absent CC
B6-*Pax9*^*–/– a*^	24	3/19 (16%)	15/19 (79%)	0	22/24 (92%)	2/24 (8%)	17/24 (71%)
CD1-*Pax9*^*–/– b*^	47	10/37 (27%)	6/37 (16%) ***	2/47 (4%)	41/47 (87%)	6/47 (13%)	27/47 (57%)
CD1-*Pax9*^*–/–*^*;Msx1*^+*/– c*^	38	2/29 (7%)	1/29 (3%)	9/38 (24%)*	11/38 (29%)***	19/38 (50%)***	9/38 (24%)*
CD1-*Pax9*^*–/–*^*;Msx1*^*–/– d*^	7	0	1/7 (14%)	0	2/7 (29%)	0	0

Ao, aorta; A-RSA, aberrant right subclavian artery; DORV + IVC, double outlet right ventricle with interventricular communication; IAA, interrupted aortic arch; VSD, perimembranous ventricular septal defect

***p < 0.001; *p < 0.05 (Fisher’s exact test for associations)

^a^Data for *Pax9*^*–/–*^ mice on a C57Bl/6J (B6) genetic background have been published [3]. Aortic arch artery defects for neonates (n = 5) and E14.5–15.5 embryos (n = 19) are pooled. VSD and DORV + IVC data from embryos only. A-RSA refers to a retro-oesophageal, cervical origin or isolated right subclavian artery. Absent common carotid artery (CC), resulting in the internal and external carotid arteries arising directly from the main aortic vessels, either unilaterally or bilaterally. All embryos had cleft palate and an absent thymus, and all embryos except *Pax9*^*–/–*^*;Msx1*^*–/–*^ had a pre-axial digit duplication

^b^For CD1-*Pax9*^*–/–*^ mice, aortic arch artery defects for neonates (n = 22) and E15.5 embryos (n = 25) are pooled. VSD and DORV + IVC data are from all embryos and n = 12 neonates (by histology)

^c^For CD1-*Pax9*^*–/–*^*;Msx1*^+*/–*^ mice, aortic arch artery defects for neonates (n = 20) and E15.5 embryos (n = 18) are pooled. VSD and DORV + IVC data from all embryos and n = 11 neonates (by histology)

^d^CD1-*Pax9*^*–/–*^*;Msx1*^*–/–*^ data for neonates (n = 1) and E15.5 embryos (n = 6) are pooled. All control B6-*Pax9*^+*/*+^ (n = 16) and CD1-*Pax9*^+*/*+^ (n = 9) embryos and neonates were normal. CD1-*Pax9*^+*/*+^*;Msx1*^*–/–*^ (n = 7) and CD1-*Pax9*^+*/–*^*;Msx1*^*–/–*^ (n = 14) embryos and neonates had normal heart, great arteries and thymus, but all had a cleft palate

*Pax9* and *Msx1* are known to be co-expressed and interact in craniofacial development [14]. To explore if *Pax9* and *Msx1* are also co-expressed in the pharyngeal arches at mid-embryogenesis we used lineage tracing and single cell analysis. To visualise specific *Pax9* and *Msx1* expression within the pharyngeal arches at E10.5, we employed two promoter specific Cre expressing lines, *Pax9*^*Cre*^ [3] and *Msx1*^*Cre−ERT2*^ [31], with the latter injected intraperitoneally with Tamoxifen the day before collection to activate Cre expression. Following X-Gal staining of whole embryos, sections were produced to examine tissue expression in more detail. This demonstrated that *Pax9* is specifically expressed in the pharyngeal endoderm at E10.5 (Fig. 2A–C). *Msx1*, on the other hand, is expressed within the pharyngeal arches in presumed NCC (Fig. 2D–F). No endoderm staining of *Msx1* was observed. To further assess the non-overlapping expression of *Pax9* and *Msx1* in the pharyngeal arches we employed single cell transcriptomics using the Fluidigm C1 System. From dissociated E9.5 caudal pharyngeal arches, the transcriptome of 86 single cells were individually analysed for gene expression (Additional file 5: Table S1). Candidate genes for each pharyngeal tissue were selected as markers for each cell type: *Pax9* for endoderm (n = 13), *Fgf8* and *Tfap2a* for ectoderm (n = 5), *Hoxa2* and *Pax3* for NCC (n = 60) and *Pecam1* and *Flt1* for endothelial cells (n = 3) (Fig. 2G). We could not, however, confidently identify any mesoderm cells in this population. We identified 25 cells expressing *Msx1* and 13 cells expressing *Pax9* (Fig. 2H). Three of these cells expressed *Msx1* and *Pax9*, but almost all *Msx1* positive cells (24/25) also expressed the NCC markers *Hoxa2* and *Pax3* (Fig. 2I). A subset of *Msx1* positive cells also expressed the smooth muscle cell markers *Pdgfrb* and *Tagln* (2/7). Collectively this data demonstrates that *Msx1* and *Pax9* do not overlap in their expression within the pharyngeal arches at mid-embryogenesis, with *Pax9* specifically expressed in the pharyngeal endoderm and *Msx1* in NCC and their derivatives.

**Fig. 3:**
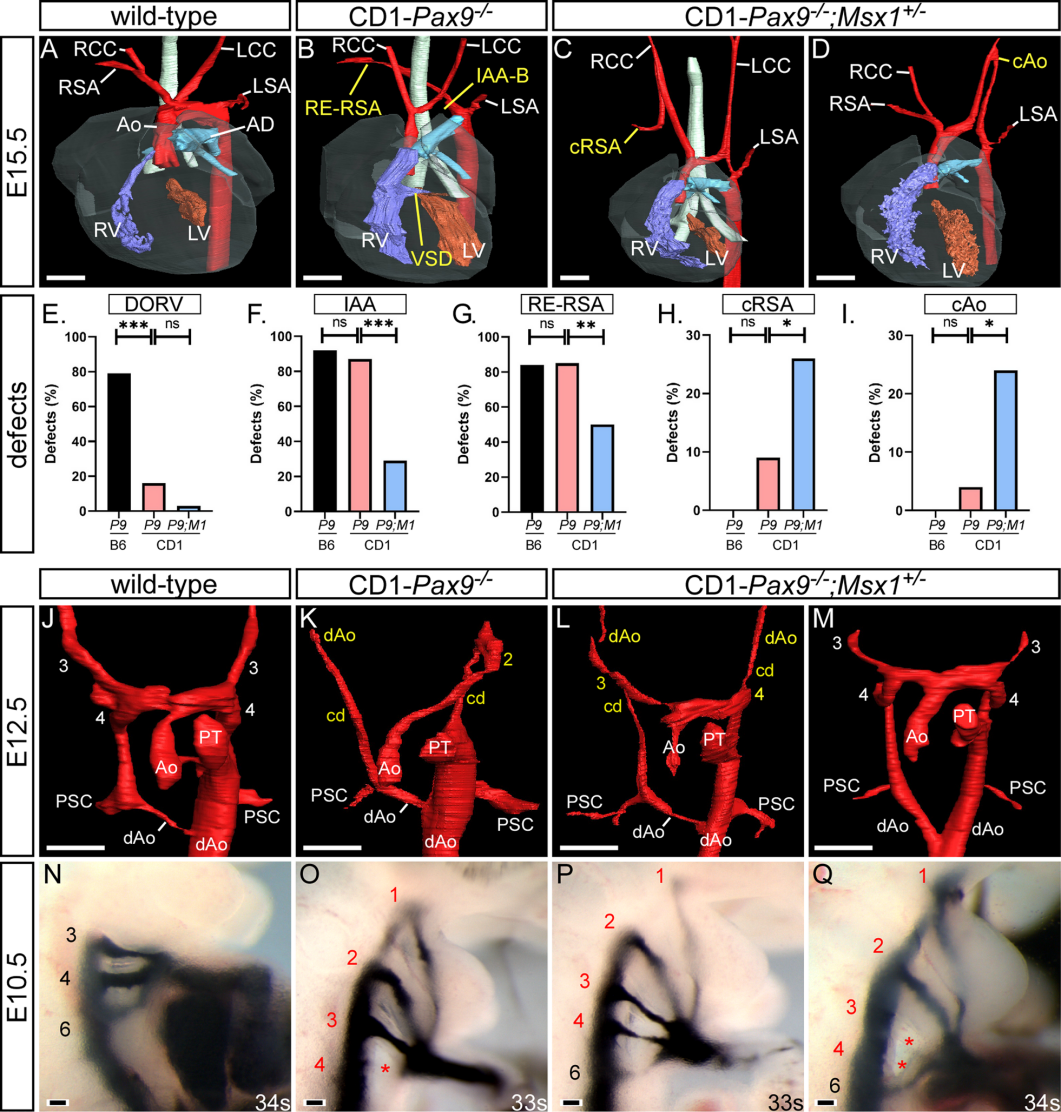
*Msx1* haploinsufficiency modifies the *Pax9*^*–/–*^ cardiovascular phenotype. Embryos were imaged by µCT at E15.5 (**A**–**D**) and E12.5 (**J**–**M**), and following intracardiac ink injection at E10.5 (**N**–**Q**). **A** Control embryo with normal heart and aortic arch artery development. **B** CD1-*Pax9*^*–/–*^ embryos have defects such as interrupted aortic arch type B (IAA-B), retro-esophageal right subclavian artery (RE-RSA) and ventricular septal defect (VSD). A proportion of CD1-*Pax9*^*–/–*^*;Msx1*^+*/–*^ embryos had cervical right subclavian artery (cRSA; **C**) or cervical aortic arch (cAo; **D**). **E**–**I** Defect frequencies seen in E15.5 embryos and neonates on C57Bl/6J (B6; data from [3]) and CD1 backgrounds are shown for *Pax9*^*–/–*^ (*P9*) and *Pax9*^*–/–*^*;Msx1*^+*/–*^ (*P9;M1*) genotypes. CD1-*Pax9*^*–/–*^ mice have a significantly reduced penetrance of outflow tract defects such as DORV (**E**) although 4th PAA-derived defects are similar (**F**, **G**). IAA-B and RE-RSA are significantly reduced in CD1-*Pax9*^*–/–*^*;Msx1*^+*/–*^ mice compared to CD1-*Pax9*^*–/–*^ mice (**F**, **G**) and cervical origins of the RSA (cRSA; **H**) and aorta (cAo; **I**) are increased. Fisher’s exact test for associations. ns, not significant; *p < 0.05, **p < 0.01, ***p < 0.001. **J** At E12.5 the aorta (Ao) and pulmonary trunk (PT) are septated and the right dorsal aorta (dAo) is regressing. **K** In CD1-*Pax9*^*–/–*^ embryos the PAA are abnormal with the 3rd and 4th PAA bilaterally absent, and the carotid duct (cd) persisting bilaterally. **L**, **M** In CD1-*Pax9*^*–/–*^*;Msx1*^+*/–*^ embryos the 3rd and 4th PAAs often appear normal. **N** Control E10.5 embryo with PAAs patent to ink. The PAAs are abnormal in CD1-*Pax9*^*–/–*^ (**O**) and CD1-*Pax9*^*–/–*^*;Msx1*^+*/–*^ (**P**, **Q**) embryos. Somite counts (s) are indicated. AD, arterial duct; LCC/RCC, left/right common carotid artery; LSA/RSA, left/right subclavian artery; LV/RV, left/right ventricle; PSC, primitive subclavian complex. Scale bars: 500 µm in A-D, 250 μm in J-M, 100 μm in N-Q

To investigate whether *Pax9* and *Msx1* functionally interact in cardiovascular development, *Pax9*^+*/–*^ and *Msx1*^+*/–*^ mice, with both lines congenic on a CD1 background, were crossed to produce compound mutant embryos and neonates for analysis. Double heterozygous mice (i.e. *Pax9*^+*/–*^;*Msx1*^+*/–*^) were viable, fertile, and phenotypically normal except for the previously recognised absence of the lower teeth [14]. *Pax9*^+*/–*^;*Msx1*^+*/–*^ mice were intercrossed to produce all possible *Pax9;Msx1* genotypes. We confirmed that *Msx1*^*–/–*^ mice on a CD1 background have cleft palate but no cardiovascular defects as previously reported [12, 19], and this was also found for the *Pax9*^+*/–*^;*Msx1*^*–/–*^ genotype (Additional file 4: Table S2). For the *Pax9*^*–/–*^;*Msx1*^+*/–*^ genotype, 18 embryos at E15.5 and 20 neonates were collected and examined, revealing a highly significant reduction in IAA-B, with or without A-RSA (*p* < 0.001) when compared with CD1-*Pax9*^*–/–*^ mice (Fig. 3A–D, F; Table 1). There was also a significant reduction in the incidence of absent common carotid arteries, a hallmark of the B6-*Pax9*^*–/–*^ cardiovascular phenotype (*p* < 0.05) [3] (Table 1), and a very low incidence of DORV (one case in 29 mutants examined; Fig. 3E). In our initial analysis, A-RSA referred to the right subclavian artery being retro-esophageal, isolated, or of a cervical origin. When this data was further analysed the incidence of retro-esophageal right subclavian artery (RE-RSA) was seen to be significantly reduced in *Pax9*^*–/–*^;*Msx1*^+*/–*^ embryos (*p* < 0.01) (Fig. 3G) and cervical origin of the right subclavian artery (cRSA) was found to be significantly increased (*p* < 0.05) (Fig. 3H). Cervical origin of the aorta (cAo) was also increased significantly (*p* < 0.05) (Fig. 3I). Wild type (n = 2), *Pax9*^*–/–*^ (n = 4) and *Pax9*^*–/–*^;*Msx1*^+*/–*^ (n = 6) embryos at E12.5 were analysed by µCT to assess the remodelling of the aortic arch arteries. This revealed that the 3rd and 4th PAAs were absent or aberrant in all *Pax9*^*–/–*^ embryos as expected [3], whereas in *Pax9*^*–/–*^;*Msx1*^+*/–*^ embryos the 3rd and 4th PAAs were maintained in 67% (8/12) and 58% (7/12) of cases, respectively (*p* < 0.05; Fig. 3J–M; Table 2).

**Table 2:** Cardiovascular defects in *Pax9:Msx1* mutant embryos at E12.5

Genotype	n	Absent 3rd PAA	Absent 4th PAA
Control	2	0/4	0/4
CD1-*Pax9*^*–/–*^	4	7/8	8/8
CD1-*Pax9*^*–/–*^*;Msx1*^+*/–*^	6*a*	4/12*	5/12*

Embryos were assessed by μCT and the 3rd and 4th PAAs (i.e. two of each per embryo) scored for being absent in each genotype. PAA defects in CD1-*Pax9*^*–/–*^*;Msx1*^+*/–*^ embryos were significantly reduced when compared to CD1-*Pax9*^*–/–*^ embryos

*PAA* pharyngeal arch artery

*p < 0.05 (Fisher’s exact test for associations)

^a^Two embryos were normal with no PAA defects

It therefore appears that mice deficient for *Pax9* and heterozygous for *Msx1* (i.e. *Pax9*^*–/–*^;*Msx1*^+*/–*^) present with alternative arch artery defects when compared to *Pax9*^*–/–*^ mice. These compound mutant mice, however, did show the other *Pax9*^*–/–*^ associated developmental defects affecting the palate, thymus and digits (as shown in Fig. 1) [4]. Overall, lack of one *Msx1* allele in the context of *Pax9* deficiency appeared to rescue the cardiovascular phenotype to a degree, with the incidence of fatal lesions, such as IAA-B, reduced and replaced with the potentially non-lethal defect of cervical origin of the aortic arch.

To further examine the effect of genetic background on the *Pax9*^*–/–*^ cardiovascular phenotype, and to also analyse the effect of *Msx1* heterozygosity, intra-cardiac ink injections were performed on CD1-*Pax9*^*–/–*^ and CD1-*Pax9*^*–/–*^;*Msx1*^+*/–*^ embryos at E10.5 to visualise the patency of the developing PAAs (Fig. 3N). Data for CD1-*Pax9*^*–/–*^ embryos (n = 9) was first compared to our published ink injected B6-*Pax9*^*–/–*^ embryos (n = 20) [3]. This analysis revealed that, like B6-*Pax9*^*–/–*^ embryos, the 4th PAAs in CD1-*Pax9*^*–/–*^ embryos were bilaterally non-patent to ink and therefore considered to be absent at this stage. The 3rd PAAs in CD1-*Pax9*^*–/–*^ embryos were also similarly affected as B6-*Pax9*^*–/–*^ embryos in the majority of cases (Fig. 3O; Table 3). Approximately half of B6-*Pax9*^*–/–*^ embryos also had aberrantly persisting 1st and/or 2nd PAAs patent to ink, and this increased to 78% (*p* = 0.41) and 89% (*p* < 0.05) respectively in CD1-*Pax9*^*–/–*^ embryos. CD1-*Pax9*^*–/–*^;*Msx1*^+*/–*^ embryos (n = 16) also displayed bilateral defects of the 4th PAAs although 37.5% (6/16) embryos had at least one vessel that was hypoplastic rather than absent, which is significantly different to CD1-*Pax9*^*–/–*^ embryos (*p* < 0.05; Fig. 3P, Q; Table 3; Additional file 1). There was a significant reduction in the incidence of 3rd PAA defects seen in CD1-*Pax9*^*–/–*^;*Msx1*^+*/–*^ embryos compared with CD1-*Pax9*^*–/–*^ embryos (25% vs. 89%; *p* < 0.005). The reduction in persistent 1st and 2nd PAAs observed, however, was not significant.

**Table 3:** Pharyngeal arch artery defects in mutant E10.5 embryos

Genetic background	n	PAA	Abnormal	Unilateral	Bilateral	Bilateral defects		
						Present	Hypo/Int/Abs	Absent
B6-*Pax9*^*–/– *a^	20	1	11 (55%)	1	10	9	1	0
		2	8 (40%)	3	5	4	1	0
		3	15 (75%)	3	12	–	8	4
		4	20 (100%)	1	19	–	3	16
CD1-*Pax9*^*–/–*^	9	1	7 (78%)	1	6	6	0	0
		2	8 (89%) *	1	7	7	0	0
		3	8 (89%)	0	8	–	3	5
		4	9 (100%)	0	9	–	0	9
CD1-*Pax9*^*–/–*^*;Msx1*^+*/–*^	16	1	8 (50%)	0	8	8	0	0
		2	8 (50%)	0	8	8	0	0
		3	4 (25%) **	0	4	–	3	1
		4	16 (100%)	0	16	–	6*	10

Embryos were collected at E10.5 and assessed for pharyngeal arch artery (PAA) defects by intracardiac ink injection

**p < 0.005, *p < 0.05 (Fisher’s exact test for associations)

^a^Data for *Pax9*^*–/–*^ embryos on a C57Bl/6J (B6) genetic background have been published [3]. For *Pax9*^*–/–*^ embryos, each left and right PAA 1–4 was scored as having a unilateral or bilateral defect, and the bilateral defects categorised as either present, a combination of hypoplastic, interrupted and/or absent (Hypo/Int/Abs), and bilaterally absent. All control B6-*Pax9*^+*/*+^ (n = 18) and CD1-*Pax9*^+*/*+^ (n = 12) embryos were normal. CD1-*Pax9*^+*/*+^*;Msx1*^*–/–*^ (n = 6) and CD1-*Pax9*^+*/–*^*;Msx1*^*–/–*^ (n = 7) embryos were normal. The increase in abnormal 2nd PAAs in CD1-*Pax9*^*–/–*^ compared with B6-*Pax9*^*–/–*^ embryos is significant. The decrease in 3rd PAA defects, and the increase in hypoplastic 4th PAA defects, in CD1-*Pax9*^*–/–*^*;Msx1*^+*/–*^ compared with CD1-*Pax9*^*–/–*^ embryos is significant

Breeding double heterozygous mice also produced double null (i.e. *Pax9*^*–/–*^;*Msx1*^*–/–*^) embryos (n = 6 at E15.5, n = 1 at E12.5, n = 2 at E10.5), and one neonate, for analysis (Additional file 2﻿; Table 1). From the mutant neonates and embryos at E15.5, two presented with IAA-B and A-RSA (Additional file 2H), and one of these also had DORV. Interestingly, *Pax9*^*–/–*^;*Msx1*^*–/–*^ mice did not have the pre-axial digit duplication seen in *Pax9*^*–/–*^ mice (Additional file 2G). Analysis of the three *Pax9*^*–/–*^;*Msx1*^*–/–*^ embryos at E12.5 and E10.5 by HREM and ink injection showed that the PAA defects were similar to *Pax9*^*–/–*^ embryos at these stages (Additional file 2L–N). The incidence of IAA-B in *Pax9*^*–/–*^;*Msx1*^*–/–*^ neonates and E15.5 embryos was the same as seen in *Pax9*^*–/–*^;*Msx1*^+*/–*^ neonates and E15.5 embryos (Table 1) but the majority of double nulls (n = 5/7; 71%) had a normal cardiovascular system at these stages.

**Fig. 4:**
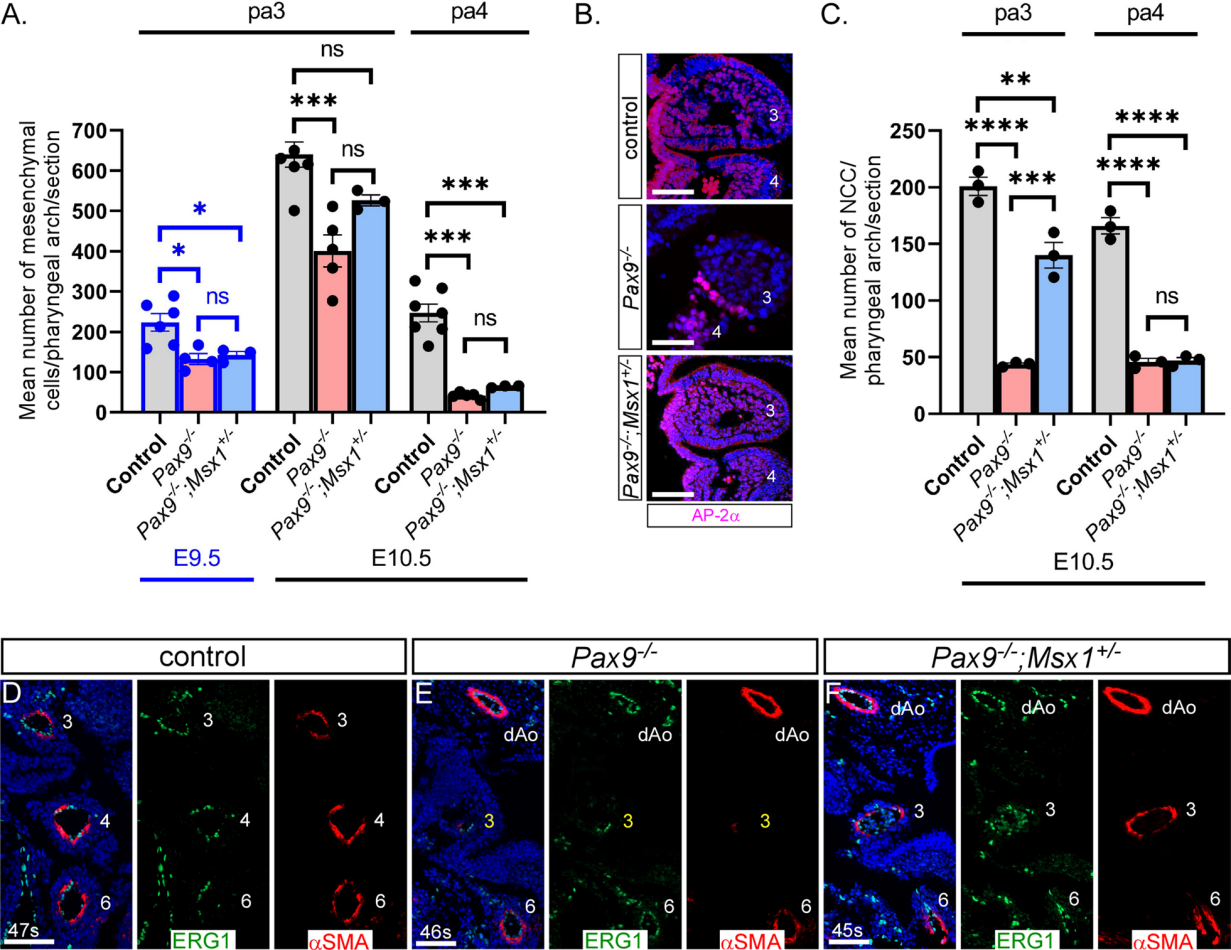
*Msx1* haploinsufficiency rescues the 3rd pharyngeal arch artery defect in *Pax9*^*–/–*^ embryos. **A** The total number of cells within the mesenchyme of the 3rd and 4th pharyngeal arches (pa) per section were counted at E9.5 and E10.5 in control (n ≥ 5), CD1-*Pax9*^*–/–*^ (n ≥ 4) and CD1-*Pax9*^*–/–*^*;Msx1*^+*/–*^ (n = 3) embryos. There were significantly fewer cells in the 3rd arch in *Pax9*^*–/–*^ and *Pax9*^*–/–*^*;Msx1*^+*/–*^ embryos at E9.5, and in the 4th arch at E10.5, compared to control. In the 3rd arch at E10.5 there were significantly fewer cells in *Pax9*^*–/–*^ embryos but the reduction in cell number was not significant in *Pax9*^*–/–*^*;Msx1*^+*/–*^ embryos. **B** Immunostaining for neural crest cells (NCC) using an anti-AP-2α antibody was performed at E10.5 (n = 3 per genotype). **C** There were significantly fewer NCC in the 3rd arch in *Pax9*^*–/–*^ and *Pax9*^*–/–*^*;Msx1*^+*/–*^ embryos when compared to control but the NCC number in *Pax9*^*–/–*^*;Msx1*^+*/–*^ embryos was significantly increased when compared with *Pax9*^*–/–*^ embryos. In the 4th arch the reduction in NCC was significant in both mutant genotypes when compared to control. One-way ANOVA with Tukey’s multiple comparisons test. ns, not significant; *p < 0.05, **p < 0.01, ***p < 0.001, ****p < 0.0001. **D**–**F** E11.5 embryo sections (n = 6 per genotype) were immunostained using an anti-αSMA antibody for smooth muscle cells (SMC) and an anti-ERG antibody for endothelium. In all control embryos, SMC surrounded the 3rd, 4th and 6th PAAs (**D**). In *Pax9*^*–/–*^ embryos the 3rd PAAs had limited recruitment of SMCs (**E**) whereas in *Pax9*^*–/–*^*;Msx1*^+*/–*^ embryos SMC were seen surrounding the 3rd PAAs (**F**). There are no 4th PAAs in *Pax9*^*–/–*^ and *Pax9*^*–/–*^*;Msx1*^+*/–*^ embryos. Somite counts (s) are indicated. Scale bars: 50 μm in B, 100 μm in D-F

To investigate if cell fate within the pharyngeal arches was affected in CD1-*Pax9;Msx1* mutant embryos, cell death and proliferation were assessed. There was no significant difference in the levels of apoptosis and proliferation between the cells of the different pharyngeal tissue layers in control, CD1-*Pax9*^*–/–*^ and CD1-*Pax9*^*–/–*^;*Msx1*^+*/–*^ embryos at either E9.5 or E10.5 (n ≥ 3 per genotype and stage) (Additional file 3). In B6-*Pax9*^*–/–*^ embryos a significantly reduced number of NCC observed in the 3rd and 4th pharyngeal arches at E10.5 has been described [3]. We firstly counted the number of cells in the pharyngeal arch mesenchyme of CD1 congenic mutant mice as this is predominantly comprised of NCC (n ≥ 3 per genotype and stage; Fig. 4A). This revealed that at E9.5 there was a significant reduction in cell number in the 3rd pharyngeal arch mesenchyme in *Pax9*^*–/–*^ and *Pax9*^*–/–*^;*Msx1*^+*/–*^ embryos compared to controls (*p* < 0.05). At E10.5 there was also a significant reduction in the number of mesenchymal cells in the 3rd pharyngeal arch of *Pax9*^*–/–*^ embryos (*p* < 0.001), but the reduction in cell number in *Pax9*^*–/–*^;*Msx1*^+*/–*^ embryos was not significantly different to controls in this tissue. In the 4th pharyngeal arch, however, there was a significant reduction in cell number in both *Pax9*^*–/–*^ and *Pax9*^*–/–*^;*Msx1*^+*/–*^ embryos (*p* < 0.001; Fig. 4A). This data, therefore, suggests that there is a reduction in NCC number in the 4th pharyngeal arch of *Pax9*^*–/–*^ and *Pax9*^*–/–*^;*Msx1*^+*/–*^ embryos, but the reduction in cell number in the 3rd pharyngeal arch of *Pax9*^*–/–*^;*Msx1*^+*/–*^ embryos at E10.5 is not significantly reduced when compared to controls. To validate this observation specifically for NCC, we immunostained coronal sections of E10.5 embryos (n = 3 per genotype) with an anti-AP-2α antibody which labels NCC (Fig. 4B). This confirmed our mesenchymal cell counting data and demonstrated that the number of NCC in the 3rd pharyngeal arch of *Pax9*^*–/–*^;*Msx1*^+*/–*^ embryos at E10.5 was significantly increased when compared with *Pax9*^*–/–*^ embryos (p < 0.001). The number of NCC in the 4th pharyngeal arch was significantly reduced in both *Pax9*^*–/–*^ and *Pax9*^*–/–*^;*Msx1*^+*/–*^ embryos when compared to controls (*p* < 0.0001; Fig. 4C).

In B6-*Pax9*^*–/–*^ embryos the reduction in SMC surrounding the 3rd PAAs was linked to the failure of this vessel to be maintained resulting in an absent common carotid artery at the foetal stage [3]. To investigate this in CD1-*Pax9*^*–/–*^ and *Pax9*^*–/–*^;*Msx1*^+*/–*^ embryos, control and mutants at E11.5 were immunostained using antibodies raised against ERG1 and smooth muscle actin for endothelium and SMC respectively (n = 6 of each genotype examined; Fig. 4D–F). This staining revealed that, like B6-*Pax9*^*–/–*^ embryos, SMC recruitment to the 3rd PAA was greatly reduced or absent in CD1-*Pax9*^*–/–*^ embryos (Fig. 4E). In *Pax9*^*–/–*^;*Msx1*^+*/–*^ embryos, however, SMC were observed surrounding the 3rd PAAs (Fig. 4F).

**Fig. 5:**
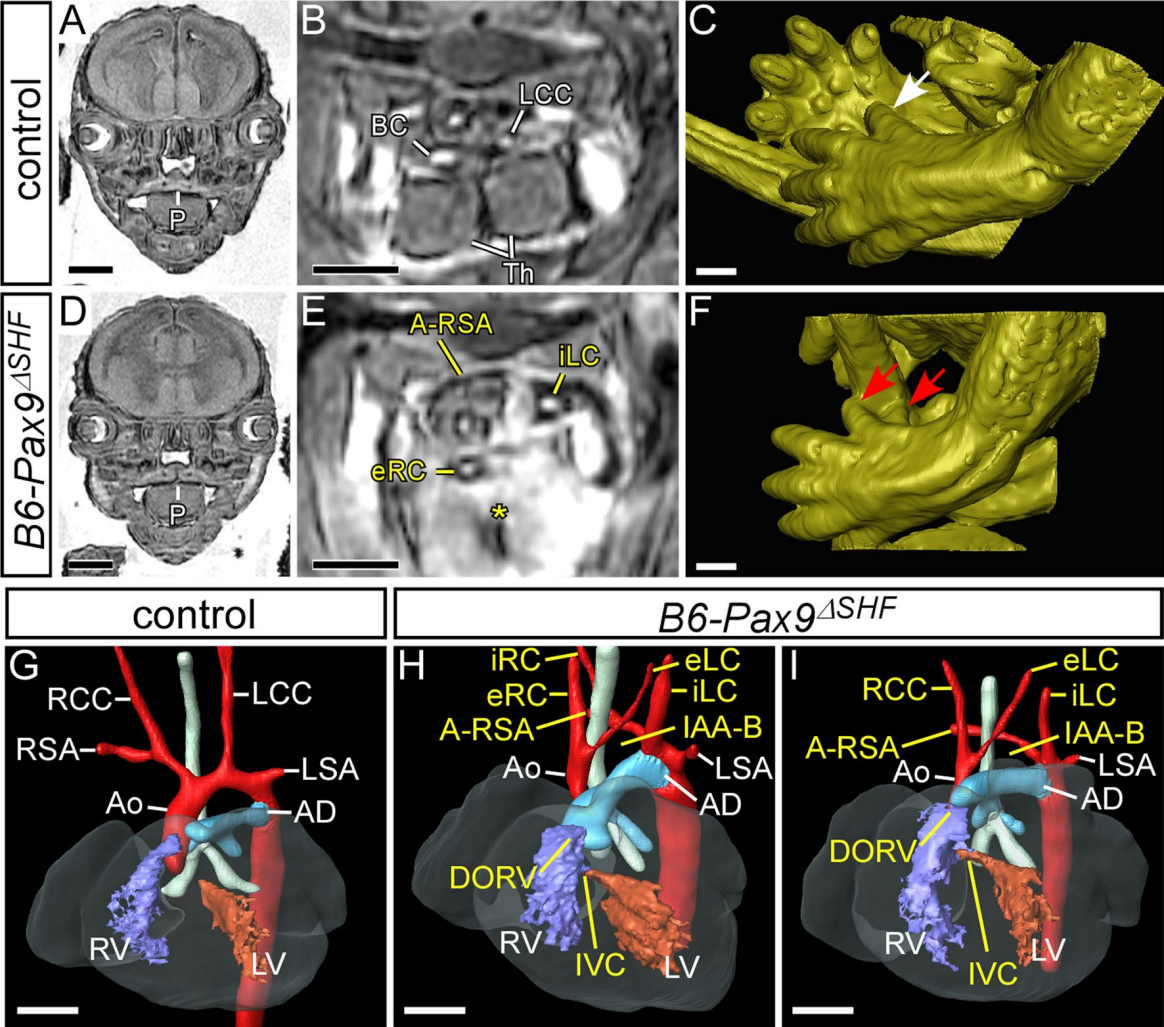
Deletion of *Pax9* from the second heart field in C57Bl/6 congenic mice recapitulates the *Pax9*^*–/–*^ cardiovascular phenotype. Embryos at E15.5 were imaged by MRI. **A**–**C** *Pax9*^+*/flox*^*;Isl1Cre* control embryos had normal development of the palate (**A**), thymus (**B**) and hind limb digit (white arrow; **C**). **D**–**F** Congenic C57Bl6/J *Pax9*^*–/flox*^*;Isl1Cre* embryos with a second heart field (SHF) conditional inactivation of *Pax9* (B6-*Pax9*^*ΔSHF*^) display a normal palate (**D**), absent thymus (asterisk; **E**) and pre-axial digit duplication (red arrows; **F**). **G**–**I** Cardiovascular defects in B6-*Pax9*^*ΔSHF*^ embryos. **G** Control embryos have normal heart and aortic arch artery development. **H**, **I** B6-*Pax9*^*ΔSHF*^ embryos present with typical *Pax9*^*–/–*^ cardiovascular defects such as double-outlet right ventricle (DORV) with interventricular communication (IVC), interrupted aortic arch type B (IAA-B), aberrant right subclavian artery (A-RSA), and absent common carotid arteries resulting in the internal and external carotid arteries (iLC, eLC, iRC, eRC) arising directly from the aorta and dorsal aorta respectively. AD, arterial duct; Ao, aorta; BC, brachiocephalic artery; LCC/RCC, left/right common carotid artery; LSA/RSA, left/right subclavian artery; LV/RV, left/right ventricle; P, palate; Th, thymus. Scale bars: 500 µm

Collectively, these data show that the reduced number of NCC in the 3rd pharyngeal arch in *Pax9*^*–/–*^ embryos is rescued to some extent in *Pax9*^*–/–*^;*Msx1*^+*/–*^ embryos. Along with the concomitant recruitment of SMC to the 3rd PAA, this suggests a developmental mechanism to explain the reduced aortic arch artery severity in mice with *Pax9* deficiency coupled with *Msx1* heterozygosity. *Pax9*^*–/–*^ mice die in the neonatal period, presumably from arterial duct-dependent defects such as IAA-B caused by failure of the left 4th PAA to form, as well as absent common carotid arteries caused by the collapse of the 3rd PAAs [3]. *Pax9*^*–/–*^;*Msx1*^+*/–*^ mice also died after birth but with a much lower incidence of IAA-B and absent common carotid arteries, and a higher occurrence of other arch artery defects such as cervical origins of the aortic arch and right subclavian artery were observed. Analysis at mid-embryogenesis showed that, although morphogenesis of the 4th PAAs was affected in all *Pax9*^*–/–*^;*Msx1*^+*/–*^ embryos, a proportion of embryos had a mildly affected 4th PAA. Moreover, there were fewer embryos with 3rd PAA defects and this was linked to the maintenance of the 3rd PAAs which were invested with SMC. We therefore hypothesised that *Msx1* heterozygosity, rather than rescuing arch artery defects, altered the type of defect to one that is apparently compatible with a functioning systemic circulation. For example, a 3rd PAA in combination with an absent left 4th PAA and a persistent carotid duct may remodel to a cervical aortic arch. *Pax9*^*–/–*^;*Msx1*^+*/–*^ mice with an apparently intact systemic circulation, however, still died soon after birth. *Pax9*^*–/–*^ and *Pax9*^*–/–*^;*Msx1*^+*/–*^ mice also have a cleft secondary palate, and we speculated that this could theoretically compromise postnatal survival independent of the cardiovascular defects [4, 7]. We therefore further hypothesised that mutant mice with a normal palate and an intact systemic cardiovascular system could survive the neonatal period following closure of the arterial duct. To engineer this configuration we used *Isl1Cre* mice [32] in conjunction with a *Pax9*-floxed allele [33]. *Isl1Cre* causes recombination in the second heart field (SHF), encompassing the pharyngeal endoderm, and the developing limb, two domains where *Pax9* is also expressed [32, 34]. We expected that the palate would not be affected in these mice as the cleft palate observed with *Pax9* deficiency is caused by a lack of expression in NCC [3, 33], a cell type with only minimal activity of *Isl1Cre* [35]. The *Pax9*^*–/flox*^*;Isl1Cre* mutant mice, hereafter referred to as *Pax9*^*ΔSHF*^, should therefore develop the typical *Pax9*^*–/–*^ cardiovascular defects but without a cleft palate. Firstly, to demonstrate the efficacy of deleting *Pax9* from the *Is1Cre* domain, we generated *Pax9*^*ΔSHF*^ mutant mice on a C57Bl/6J genetic background. From nine *Pax9*^*ΔSHF*^ embryos at E15.5 examined by MRI, all presented with the typical *Pax9*^*–/–*^ phenotype, affecting the cardiovascular system, thymus and digit formation, although the palate was normal as predicted (Fig. 5; Table 4). There was no significant difference from the cardiovascular defects observed in B6-*Pax9*^*–/–*^ mice.

**Table 4:** Cardiovascular defects in second heart field mutant embryos and neonates

Genetic background—genotype and stage	n	VSD	DORV + IVC	cAo	IAA-B ± A-RSA	A-RSA	Absent CC
B6-*Pax9*^*ΔSHF*^ E15.5	9	1/9 (11%)	5/9 (56%)	1/9 (11%)	8/9 (89%)	1/9 (11%)	9/9 (100%)
CD1-*Pax9*^*ΔSHF*^ Neonate	1	N/A	N/A	0	1	0	1
CD1-*Pax9*^*ΔSHF*^*;Msx1*^+*/–*^ Neonate	8	0	0	2/8 (25%)	0	2/8 (25%)	0

All mice with *Pax9* conditionally inactivated from the second heart field with *Isl1Cre* (*Pax9*^*ΔSHF*^) had pre-axial digit duplication, absent thymus and normal palate. N/A, not assessed

A-RSA, aberrant right subclavian artery; cAo, cervical aorta; CC, common carotid artery; DORV + IVC, double outlet right ventricle with interventricular communication; IAA,-B interrupted aortic arch type B; SHF, second heart field; VSD, perimembranous ventricular septal defect

**Fig. 6:**
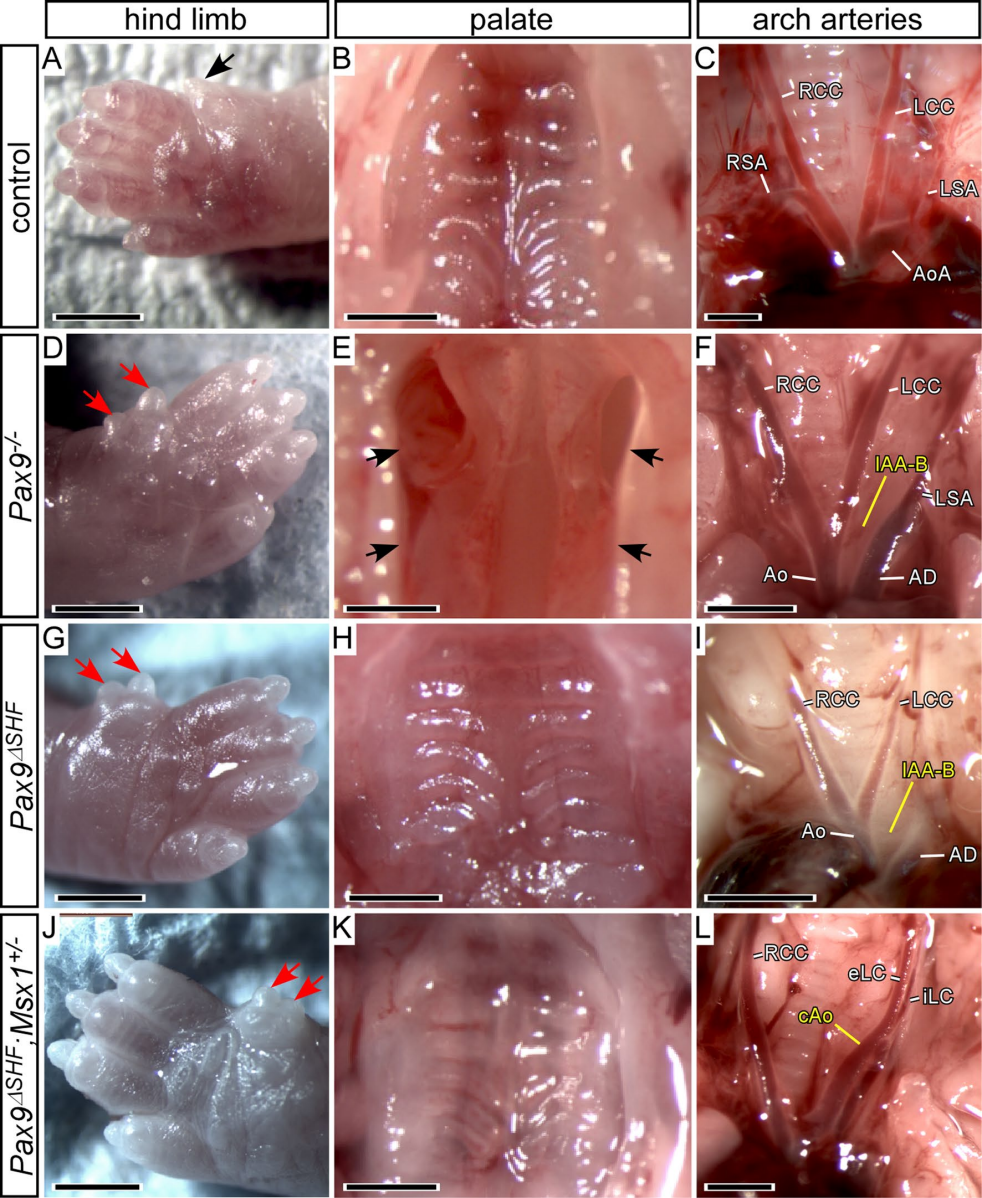
Deletion of *Pax9* from the second heart field in neonates on an enriched CD1 background. **A**–**C** *Pax9*^+*/flox*^*;Isl1Cre* control neonates had normal hind limb digit (black arrow; **A**), palate (**B**) and arch artery (**C**) development. **D**–**F** *Pax9*^*–/–*^ neonates from this cross displayed the typical *Pax9*^*–/–*^ phenotype of pre-axial digit duplication (red arrows; **D**), cleft palate (black arrows; **E**) and cardiovascular defects such as interrupted aortic arch type B (IAA-B; **F**). **G**–**I** Only one neonate with *Pax9* conditionally inactivated from the second heart field (*Pax9*^*ΔSHF*^) was recovered but showed a pre-axial digit duplication (red arrows; **G**), a normal palate (**H**) and IAA-B (**I**). **J**–**L** *Pax9*^*ΔSHF*^*;Msx1*^+*/–*^ neonates displayed a pre-axial digit duplication (red arrows; **J**), a normal palate (**K**) and frequently mild arch artery defects such as cervical aortic arch (cAo) and abnormal external and internal left common carotid artery (eLC, iLC; **L**). Ao, aorta; AoA, aortic arch; CP, cleft palate; LCC/RCC, left/right common carotid artery; LSA/RSA, left/right subclavian artery. Scale bars: 1 mm

We next generated *Pax9*^*ΔSHF*^ and *Pax9*^*ΔSHF*^*;Msx1*^+*/–*^ neonates for analysis on a CD1 genetic background. Due to the complex mating scheme required to generate mutant mice we only collected neonates so as to conserve the dams for subsequent breeding. All neonates that were found dead soon after birth were collected and analysed for arch artery defects, cleft palate and pre-axial digit duplication. All surviving neonates were culled five days after birth and examined the same way. Genotyping revealed that all neonates found dead on the day of birth had a *Pax9*^*–/–*^ genotype. All control genotypes analysed (n = 5) had a normal palate and arch arteries (Fig. 6A–C). All *Pax9*^*–/–*^ neonates (n = 5) presented with IAA-B and A-RSA as well as a cleft palate and pre-axial digit duplication (Fig. 6D–F). From two *Pax9*^*–/–*^;*Msx1*^+*/–*^ neonates recovered from this cross, both had a cleft palate and pre-axial digit duplication, one had a cervical right subclavian artery, and the other a cervical aortic arch. Only one *Pax9*^*ΔSHF*^ neonate was recovered, but showed the expected arch artery defects (IAA-B and A-RSA) and pre-axial digit duplication seen in *Pax9*^*–/–*^ mice, although the palate was normal (Fig. 6G–I; Table 4). Eight neonates with the *Pax9*^*ΔSHF*^*;Msx1*^+*/–*^ genotype (i.e. *Pax9* deleted from the SHF in conjunction with *Msx1* heterozygosity) were recovered, all found dead on the day of birth. The pre-axial digit duplication was seen, but the palate was unaffected, and the aorta and right subclavian artery were either normal (n = 6) or of cervical origin (n = 2; Fig. 6J–L; Table 4). Histology confirmed that the outflow tract, arterial valves and ventricular septum were normal. The cardiovascular system of *Pax9*^*ΔSHF*^*;Msx1*^+*/–*^ neonates was therefore normal or had a phenotype theoretically compatible with a functioning systemic circulation, and an unaffected palate, yet these mice died on the day of birth.

**Fig. 7:**
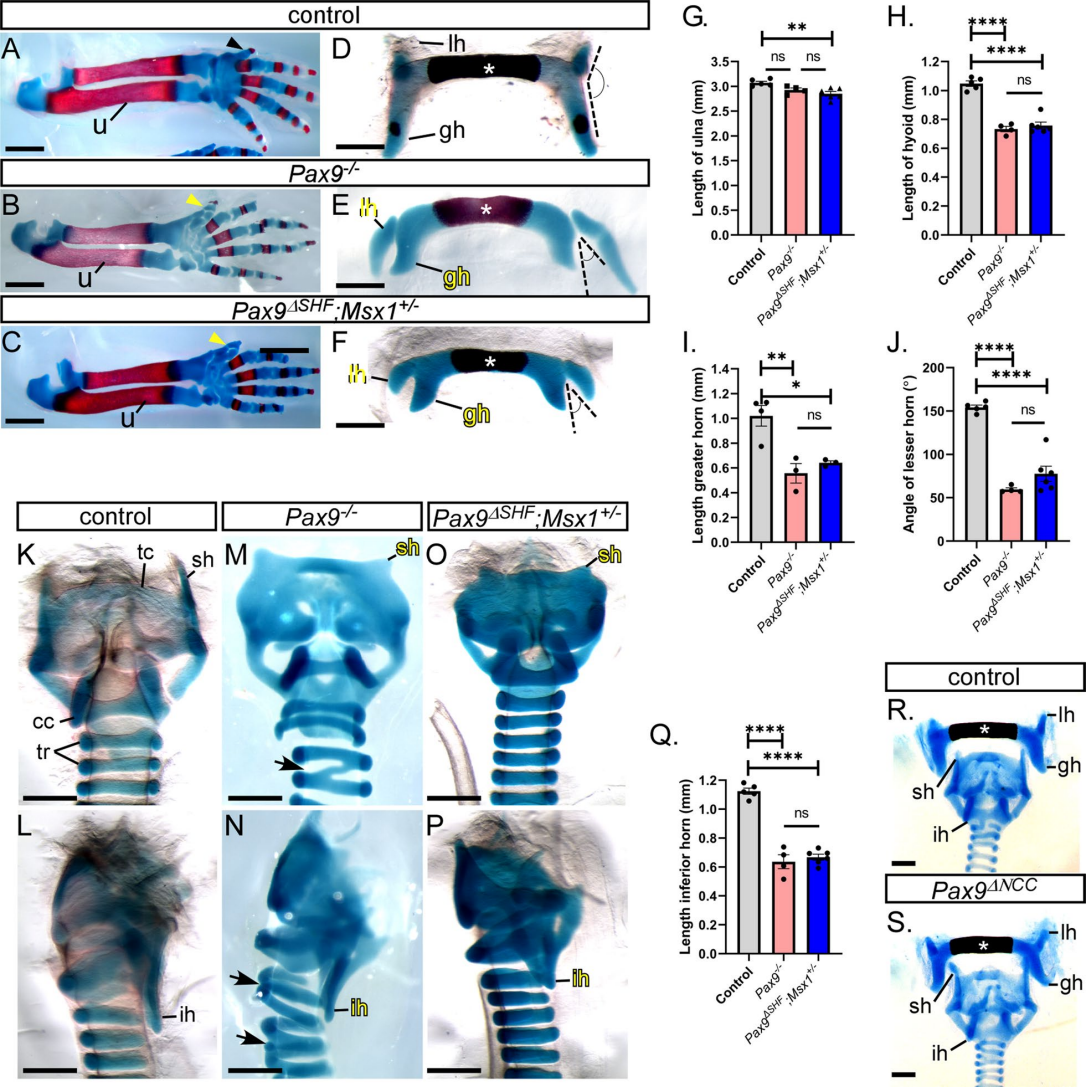
Skeletal defects in *Pax9* mutant mice on an enriched CD1 background. Neonate skeletons were stained for cartilage (blue) and bone (red). **A**–**C** Forelimbs in control neonates were normal (**A**), but a pre-axial digit duplication was present in *Pax9*^*–/–*^ (**B**) and *Pax9*^*ΔSHF*^*;Msx1*^+*/–*^ (**C**) neonates (yellow arrowhead). **D**–**F** Skeletons were disarticulated to isolate the hyoid bone. **G** The ulna (u in **A**–**C**) of control (n = 5), *Pax9*^*–/–*^ (n = 4) and *Pax9*^*ΔSHF*^*;Msx1*^+*/–*^ (n = 6) neonates was measured. There was a small yet significant reduction in the average length of the ulna in *Pax9*^*ΔSHF*^*;Msx1*^+*/–*^ neonates. **H**–**J** The ossification centre of the hyoid bone (asterisk in **D**–**F**), the length of the greater horn (gh) and the angle between the lesser (lh) and greater horns, were measured. **K**–**P** The tracheal rings (tr), thyroid (tc) and cricoid cartilages (cc) were examined. The superior horn (sh) of the thyroid cartilage was greatly reduced in size in *Pax9*^*–/–*^ and *Pax9*^*ΔSHF*^*;Msx1*^+*/–*^ neonates and the inferior horn (ih) was significantly reduced in length (**Q**). Fused tracheal rings were observed in *Pax9*^*–/–*^ neonates (arrows; **M**, **N**). **R**, **S** The hyoid and thyroid cartilage were normal in neonates with a conditional inactivation of *Pax9* from neural crest cells (*Pax9*^*ΔNCC*^). One-way ANOVA with Tukey’s multiple comparisons test. ns, not significant; **p < 0.01, ****p < 0.0001. Scale bars: 1 mm in **A**–**C**, 500 µm in **E**–**G**, **J**–**O**, **R**, **S**

As *Pax9* deficiency has been shown to cause bone malformations such as pre-axial digit duplication and cleft palate [4] we investigated the skeletons of control, *Pax9*^*–/–*^ and *Pax9*^*ΔSHF*^*;Msx1*^+*/–*^ neonates to see if any phenotype here could explain the neonatal death. Neonatal skeletons were stained for cartilage and bone using alcian blue and alizarin red. All control neonates (n = 5) had a normal skeleton (Fig. 7A). The *Pax9*^*–/–*^ (n = 4) and *Pax9*^*ΔSHF*^*;Msx1*^+*/–*^ (n = 6) skeletons all presented with a pre-axial digit duplication on the hind and forelimbs (Fig. 7B, C) as expected. The ulna length for each line was measured and showed there was little difference in neonate size between the genotypes (Fig. 7G). In control neonates the normal hyoid bone, which connects to various ligaments and muscles such as the thyrohyoid and stylohyoid ligaments [36], had a horseshoe-shape, with an elongated and flat body, and two pairs of greater and lesser horns which projected posteriorly and anteriorly, respectively, from the outer borders of the body (Fig. 7D). In the *Pax9*^*–/–*^ and *Pax9*^*ΔSHF*^*;Msx1*^+*/–*^ mutants, the ossification centre of the body of the hyoid bone was significantly shorter compared to controls (*p* < 0.0001; Fig. 7E, F, H), the lesser horn extended laterally and the greater horn was significantly reduced in length (Fig. 7E, F, I). The angle between the greater and lesser horns was significantly reduced in *Pax9*^*–/–*^ and *Pax9*^*ΔSHF*^*;Msx1*^+*/–*^ neonates (*p* < 0.0001) when compared to controls (Fig. 7E, F, J). *Pax9* deficiency also causes thyroid cartilage deformities, where the thyroid cartilage is broader and lacks the lateral processes normally connecting the thyroid and cricoid cartilages [4]. Control neonates showed a normal thyroid cartilage with two laminae that fused together anteriorly (Fig. 7K, L). The posterior border of each lamina was free and created the superior and inferior horn projections. In *Pax9*^*–/–*^ and *Pax9*^*ΔSHF*^*;Msx1*^+*/–*^ neonates the inferior horn of the thyroid cartilage was significantly shorter compared to controls, and the superior horn was reduced to a stump (Fig. 7M–Q). Fused tracheal rings were also observed in *Pax9*^*–/–*^ neonates (Fig. 7M, N). The hyoid bone and thyroid cartilage are both derived from NCC [37, 38] but neonates with a conditional inactivation of *Pax9* from NCC, however, did not have any hyoid bone or thyroid cartilage defects (n = 11; Fig. 7R, S).

## Discussion

In this study we have shown that mice deficient for *Pax9* on a congenic CD1 background, compared to those congenic for C57Bl6/J [3], have a significantly reduced incidence of DORV and do not show bicuspid aortic valve or hypoplastic aorta. The incidence of aortic arch defects, however, are very similar. We have also shown that *Pax9*^*–/–*^ mice heterozygous for *Msx1* have a significantly reduced incidence of arterial duct-dependent defects but an increase in alternative arch artery defects that could have been compatible with post-natal life in the absence of a cleft palate. Mice with this phenotype, however, did not survive after birth and most likely died from *Pax9*-deficiency causing non-cell autonomous defects to the hyoid bone and thyroid cartilage.

It is well known that changing the genetic background in mouse models of disease may influence the presentation of specific phenotypes due to strain-specific modifiers [27]. For example, *Cited2*-deficient mice congenic for C57Bl6/J had left–right patterning defects [29] that were not apparent on a mixed genetic background [39]. On a Swiss Webster background mice heterozygous for *Tbx1* do not show the typical 4th PAA derived arch artery defects [40], and arch artery defects change according to genetic background in *Ap2a*-null embryos [28]. It is difficult to speculate on a mechanism to explain how the change in the outflow tract phenotype in Pax9^*–/–*^ mice on a different genetic background is controlled. Given that outflow tract defects are typically caused by issues with the SHF [41] it is possible that genetic modifiers on the CD1 background are protective of the SHF, allowing it to proceed with development almost normally. Deletion of *Tbx1* results in severe cardiovascular defects affecting the outflow tract and arch arteries [42] but the conditional reactivation of *Tbx1* expression in the SHF mesoderm is able to rescue the OFT defects, but not the 4th PAA defects [43]. This indicates that there is a complex regulation in gene expression within the pharyngeal region that controls different aspects of cardiovascular development. Tbx1 is a DNA binding transcription factor that has many functions, including chromatin remodelling, histone modification and priming enhancers for downstream activation or repression [1, 44]. *Tbx1* functionally interacts with *Pax9* [3], which also has additional roles beyond being a transcription factor as a regulator of heterochromatin [45] and in ribosome production [46]. There are therefore multiple areas for targeting by background-specific modifiers that may affect cardiovascular defects caused by *Pax9* deficiency.

Future studies could employ quantitative trait loci analysis to detect important chromosomal linkage markers to identify modifier regions that would explain the genetics underlying these phenotypic changes. Patients with heterozygous frameshifts, nonsense and missense mutations in *PAX9* commonly present with non-syndromic hypodontia or oligodontia [47–51], but although reports of *PAX9* deletions are infrequent, some do include heart defects [23–26]. This heterogeneity in phenotype presentation may be an example of genetic or environmental modifiers which have a detrimental effect on cardiovascular development.

During cardiovascular development the PAAs form symmetrically and rapidly remodel to become the asymmetric arch arteries seen in the adult. Specifically, the 3rd PAAs form the common carotids and the proximal part of the internal carotid arteries, and the 4th PAAs form the aortic arch on the left and the proximal part of the subclavian artery on the right [52, 53]. The section of the paired dorsal aorta between the 3rd and 4th PAAs, the carotid duct, involutes and the dorsal aorta cranial to this becomes the distal internal carotid artery. In patients, failure of the 3rd PAAs to be maintained results in the absence of the common carotid arteries and the separate origins of the internal and external carotids [54], a phenotype seen in *Pax9*^*–/–*^ mice [3]. This defect is often seen in conjunction with a cervical aortic arch [55] which is clinically rare but may be asymptomatic [56], and is associated with 22q11 Deletion Syndrome (DS) [57]. If the right 4th PAA does not form, an A-RSA is seen, which may be retro-esophageal or cervical in origin. RE-RSA is considered a variant as it is relatively common, seen in ~ 1% of the population [58], but is more prevalent in Down syndrome [59] and may be asymptomatic or cause difficulties in swallowing [60]. In mice, heterozygous deletion of *Tbx1*, the gene associated with the cardiovascular defects in 22q11DS patients [42, 61, 62], predominantly affects development of the 4th PAAs and up to half of these mutants have RE-RSA but they are viable [61]. If the left 4th PAA fails to form, however, this results in an IAA-B which is lethal once the arterial duct closes postnatally [60], and half of all cases of IAA-B are found in 22q11DS patients [63–65]. IAA-B is only observed in a minority of mice heterozygous for *Tbx1* although when combined with *Pax9* heterozygosity this increases considerably suggesting that *Tbx1* and *Pax9* functionally interact in 4th PAA morphogenesis [3]. *Pax9*^*–/–*^ mice display a high penetrance of IAA-B, but in *Pax9*^*–/–*^;*Msx1*^+*/–*^ mutants the incidence of this defect is significantly reduced and there is an increase in potentially non-lethal arch artery defects such as cervical origins of the aortic arch and right subclavian artery. These defects have been reported in a child with IAA in which the explanation for the patient’s survival was the presence of these cervical vessels that allowed for a complete systemic circulation through the remodelled 3rd PAAs in combination with the persistence of the carotid ducts [66].

From our data it appears that *Msx1* heterozygosity in *Pax9*-deficient mice enables the normal development of the left and right 4th PAAs in a proportion of mutants as shown by a significant reduction in the incidence of IAA and RE-RSA, respectively. Immunostaining for SMC, and counting NCC in the pharyngeal arches, at mid-embryogenesis upholds the idea that the 3rd PAAs are stabilised in *Pax9*^*–/–*^;*Msx1*^+*/–*^ mutants by NCC migration and differentiation into SMC to support the vessels as they morph into the common carotid arteries. This does not occur in mice deficient for *Pax9* where the 3rd PAAs collapse leading to absence of the common carotid arteries and an arterial duct-dependent defect [3]. We have confirmed here that *Pax9* and *Msx1* have non-overlapping expression patterns in the pharyngeal arches at mid-embryogenesis, with *Pax9* specifically expressed in the pharyngeal endoderm, and *Msx1* in NCC. Interestingly, it has recently been shown that Pax9 is expressed in adult aortic vascular SMC, and *Pax9* siRNA knockdown in vitro inhibits their phenotypic transformation, proliferation and migration [67]. It is therefore possible, at least in adult aorta, that PAX9 may have a cell autonomous role in SMC. Ink injection data at E10.5, and μCT analysis at E12.5, confirms that the morphogenesis of fewer 3rd and 4th PAAs are affected in the *Pax9*^*–/–*^;*Msx1*^+*/–*^ mutants compared to *Pax9*^*–/–*^ mice. It is well recognised, particularly in *Tbx1*-heterozygous mice, that the 4th PAAs have the capacity to recover from a hypoplastic vessel to a normal arch artery during development [3, 68, 69]. As a significantly higher proportion of hypoplastic 4th PAAs are seen in *Pax9*^*–/–*^;*Msx1*^+*/–*^ compared to *Pax9*^*–/–*^ mutants, this could lead to more normal aortic arches or right subclavian arteries being formed in later development. In the absence of 4th PAA derived aortic arch arteries, and in conjunction with a significantly increased proportion of intact 3rd PAAs in *Pax9*^*–/–*^;*Msx1*^+*/–*^ embryos, we propose that these remodel to form an aorta or right subclavian artery of cervical origin with persistent carotid ducts. In theory this phenotype should have been compatible with an intact systemic circulation and neonatal survival, but this was not the case. We therefore considered that the cleft palate seen in all neonates with a *Pax9*^*–/–*^ genotype may be causing the neonatal lethality as mutant mice with cleft palate die within 24 h of birth [10]. We used conditional inactivation of *Pax9* in the SHF to engineer mutants which recapitulated the cardiovascular and limb defects of the *Pax9*^*–/–*^ mice but did not affect development of the palate. These mutants, nevertheless, did not survive long after birth despite the majority having a cardiovascular phenotype that could theoretically support a systemic circulation. Analysis of the neonatal skeletons, however, revealed defects of the hyoid bone and thyroid cartilage. The hyoid bone attaches to many muscles and ligaments associated with the floor of the mouth, as well as the larynx, pharynx, tongue, and epiglottis. The hyoid bone is necessary to maintain patency of the airway between the oropharynx and tracheal rings, and also functions in swallowing and breathing, as well as maintaining the posture of the head [70]. Given the importance of the hyoid bone in swallowing, malformation is a likely candidate for neonatal death, especially when considering the *Pax9*^*–/–*^ phenotype, which includes gasping respiration with redirection of air into the intestines, lack of suckling and presence of milk in the stomach, and the absence of a lung phenotype [4]. Also, it has been reported that muscle abnormalities produce a similar defect and the hyoid bone coordinates muscles involved in swallowing [10]. Additionally, fracture of the hyoid bone may severely compromise the upper airway [71]. *Pax9* has been shown to functionally interact with *Tbx1* in the pharyngeal endoderm [3] and mutant mice, in which *Tbx1* was deleted specifically from NCC have a hyoid defect and die in the neonatal period with no remarkable craniofacial or other phenotypes that could explain death [72]. Interestingly, the neonates show the same absence of feeding as *Pax9* mutants [4]. Furthermore, conditional deletion of *Tbx1* with *Twist2-Cre* (described as being active in osteochondro-progenitors) produces a similar hyoid phenotype and the mutants die on the day of birth without feeding and without any other noticeable defects that could indicate another cause of death [72]. The shortened region of hyoid ossification seen in our study, however, is unlikely to be the cause of death as a similar phenotype is seen in *Runx2* heterozygous mice, and these are viable [73]. The structural abnormalities seen in the horns of the hyoid and thyroid cartilage in *Pax9* mutant mice are therefore more likely to be the cause of death through breathing and feeding difficulties, as well as vocalisations, all of which are detrimental to the survival of the neonate [10].

*Pax9* is expressed in cranial NCC, and mutants with a conditional inactivation using the *Wnt1Cre* transgene develop a cleft palate but they do not have any cardiovascular abnormalities [3, 33]. The lesser horns of the hyoid are derived from NCC populating the second pharyngeal arch, the greater horns and body of the hyoid are from the third, and throat cartilage from the fourth pharyngeal arch NCC [74, 75]. Here we now show that loss of *Pax9* from NCC does not affect the hyoid bone or thyroid cartilage structures. It is therefore likely that the defects of these structures in *Pax9*^*–/–*^ and P*ax9*^*ΔSHF*^ mutants originate from loss of *Pax9* expression in the endoderm. This could be analogous to *Tbx1* inactivation from the pharyngeal endoderm which leads to aplasia of the tympanic ring, a structure derived from non-Tbx1 expressing mesenchyme [76]. Although the mechanism as to how this occurs has not been defined, it is feasible that a non-cell autonomous signalling event from the endoderm, involving *Pax9*, has been perturbed. It is known that signals from the endoderm regulate neural crest patterning [77, 78]. A mechanism such as this could explain why there is a reduction in the number of pharyngeal arch NCC and a loss of SMC supporting the remodelling 3rd PAAs in *Pax9*^*–/–*^ embryos [3]. An alternative explanation, however, is that a structural defect in the pharyngeal endoderm itself may be affecting NCC behaviour. The caudal pharyngeal arches of *Pax9*^*–/–*^ embryos are smaller than in wild-type embryos [3, 4] suggesting that the structure of this tissue itself may play a role in the overall phenotype. Studies have implicated the chicken and zebrafish endoderm in exhibiting a signalling interaction with NCC to influence their morphogenetic potential in the formation of the hyoid [79, 80]. Furthermore, it has been shown that Fibroblast Growth Factor-dependent morphogenesis of the pharyngeal endoderm into pouches in zebrafish is critical for the later patterning of the hyoid cartilages [81].

The mechanism underlying the effect of *Msx1* heterozygosity on the *Pax9*^*–/–*^ phenotype still needs to be deduced but could be related to signalling processes between the *Pax9*-expressing endoderm and *Msx1*-expressing NCC. The mechanism, however, appears to involve the rescue of NCC migration and differentiation to the smooth muscle cells that invest the remodelling third PAAs. This presumably stabilises the vessels enabling them to complete their morphogenesis into the common carotid arteries. Future work to uncover the genetic interactions controlling this process could examine the expression of key genes linked to *Pax9* and *Msx1*. Although *Pax9* and *Msx1* have been shown to regulate Bmp4 in craniofacial development [16], and loss of *Bmp4* results in PAA defects [82] we did not detect any change in *Bmp4* expression in *Pax9*^*–/–*^ pharyngeal arches at E9.5 in our published RNA-seq data [3]. This suggests that alternative interacting genes need to be investigated such as Notch ligands and their downstream effectors (e.g. *Hes*, *Hey*), which are also known to play crucial roles in cardiovascular development [83].

Furthermore, *Pax9*^*–/–*^ mice have a duplicated first digit [4], whereas *Msx1;Msx2* double null mutants either lack this digit or form an additional one [84]. In our *Pax9*^*–/–*^;*Msx1*^*–/–*^ double knockout mutants the digits are normal, indicating that complete loss of *Msx1* can further modulate the presentation of a phenotype in tissues other than those that form the cardiovascular system. All *Pax9*^*–/–*^ and *Pax9*^*–/–*^*;Msx1*^+*/–*^ mutants have some form of aortic arch artery defect at the foetal and neonatal stages, but of the *Pax9*^*–/–*^*;Msx1*^*–/–*^ double nulls examined five out of seven had normal cardiovascular development with no apparent defects of the heart or arch arteries. Although conclusions about PAA development at the younger stages are difficult to make because of the low numbers of mutants collected at E10.5 and E12.5, it does seem likely that a rescue of the *Pax9*^*–/–*^ cardiovascular phenotype through loss of both *Msx1* alleles has occurred. This, coupled with the absence of a limb phenotype in the double homozygous mutants, strongly suggests a complex regulation of gene expression controlled by *Pax9* and *Msx1*. Further experiments will be required to understand how these two genes are interacting in a gene regulatory network that is critical for embryonic development.

## Conclusions

Mice deficient for *Pax9* on a CD1 congenic background present with different cardiovascular defects when compared to mice congenic for C57Bl/6. Although it is well known that genetically altered mice may present with different phenotypes depending on the genetic background they are bred or maintained on, this study demonstrates the need to establish the full phenotype on any new genetic background being used, particularly when making comparisons investigating interactions between two genes where the mouse strains may be different.

We have shown that *Msx1* haploinsufficiency in *Pax9*^*–/–*^ mice changes the arch artery phenotype through an unknown mechanism which involves the partial rescue of NCC migration to the 3rd pharyngeal arches and the stabilisation of the 3rd PAAs by investment with SMC. We propose that in the absence of 4th PAAs, the 3rd PAAs, with persistence of the carotid ducts, are able to remodel into an aortic arch or right subclavian artery of cervical origin theoretically compatible with a systemic blood circulation. Despite engineering this phenotype in mice, but without a cleft palate, neonatal death still occurred, and this was subsequently found to be possibly caused by defective hyoid bone and thyroid cartilage development via a putative Pax9 and/or endodermal non-cell autonomous signalling pathway.

## Materials and methods

### Mice

The mice used in this study have previously been described elsewhere: *Pax9*^+*/–*^ [4], *Pax9*^*flox*^ [33], *Pax9*^*Cre*^ [3], (the *Pax9* lines were created in our laboratories), *Msx1*^+*/–*^ [12] (acquired from Richard Maas, Brigham Women’s Hospital, Boston, MA, USA), *Msx1*^*Cre−ERT2*^ [31] (acquired from Benoit Robert, Institut Pasteur, Paris, France), *Isl1Cre* [32] (acquired from Sylvia Evans, University of California San Diego, CA, USA), *Wnt1Cre* [85] (acquired from Andrew McMahon, University of Southern California, CA, USA) and *R26R*^*lacZ*^ [86], (purchased from The Jackson Laboratory, Bar Harbor, ME, USA). All mice were maintained on a congenic CD1 genetic background unless otherwise stated. All studies involving animals were performed in accordance with UK Home Office Animals (Scientific Procedures) Act 1986 and in compliance with the ARRIVE guidelines.

### Breeding

Male and female mice were mated and the detection of a vaginal plug the next morning considered to be embryonic day (E) 0.5. Pregnant females were either culled on the required day and embryos collected or allowed to litter for collection of neonates. All mice used in the study were euthanised by cervical dislocation. Embryos at E9.5-E11.5 were staged by somite counting. All mouse lines and embryos were genotyped by standard PCR (primer details given in Additional file 4﻿: Table S3).

### Imaging

Magnetic resonance imaging (MRI), micro-computed tomography (µCT) and high resolution episcopic microscopy (HREM) techniques were performed as previously described [3, 87–90]. To visualise patency of pharyngeal arch arteries at E10.5, embryos were injected with India ink via the left ventricle with pulled Pasteur pipettes [3].

### Histology, X-Gal staining and Immunohistochemistry

Whole embryos and neonate hearts were processed for histology and immunostaining analysis. Briefly, samples were fixed in 4% paraformaldehyde, dehydrated in ethanol and embedded in paraffin wax blocks for sectioning at 8 μm thickness using a Leica RM 2235 microtome (Leica Biosystems, Milton Keynes, UK). For histology, slides were dewaxed, rehydrated, and stained with haematoxylin and eosin (ThermoFisher Scientific, Waltham, MA, USA) using a standard protocol, and mounted with histomount (National Diagnostics, Atlanta, GA, USA). X-Gal staining to visualise *lacZ* expression at E10.5 was performed using standard techniques. To activate Cre expression in pregnant *Msx1*^*Cre−ERT2*^ mice, Tamoxifen (3 mg) was administered intraperitoneally at E9.5 as described [18]. For immunohistochemistry, slides with coronal sections of each embryo were dewaxed, rehydrated and immunostained with the primary antibodies shown in Additional file 4: Table S4. To assess an apoptotic index within the pharyngeal arches, control and mutant embryos at E9.5 and E10.5 (n = 3 per genotype) were examined following immunostaining with the anti-caspase 3 antibody. To assess a proliferative index within the pharyngeal arches, control and mutant embryos at E9.5 and E10.5 (n = 3 per genotype) were examined following immunostaining with the anti-histone H3 antibody. Nuclei were stained with DAPI. Each pharyngeal arch examined was demarcated by the flanking pouch and cleft of the anterior and posterior arch, and cells from n ≥ 3 sections per embryo were counted. All stained slides were viewed with a Zeiss Axioplan microscope equipped with Axiovision software (Carl Zeiss, Jena, Germany). The apoptotic or proliferative index was calculated by counting the number of positively stained cells divided by the total number of DAPI stained cells within each pharyngeal arch. To count neural crest cells, sections were immunostained using the anti-AP-2α antibody and positively stained cells within each pharyngeal arch counted (n = 3 per genotype; n ≥ 3 sections per embryo). Cell counting was performed using ImageJ software (National Institutes of Health, Bethesda, MD, USA) using the cell counter feature.

### Bone and cartilage staining

Bone and cartilage staining was performed on the skeletons of postnatal day 0 (P0) neonates found dead on the day of birth, or euthanised at P5 using 200 mg/ml of pentobarbital sodium solution (Euthatal; Boehringer Ingelheim Animal Health UK Limited, Bracknell, UK). Neonates were incubated at 70ºC for 30 s, the skin peeled away and eviscerated, fixed in 95% ethanol overnight at room temperature and then transferred to acetone at room temperature for 48 h to remove the fat. The neonates were then rinsed in water and incubated in 0.01% alcian blue solution (Merck Life Science UK Limited, Gillingham, UK) for 8 days to stain the cartilage. The neonates were washed with 70% ethanol and incubated in 1% potassium hydroxide solution until the tissue was visibly cleared. Staining of bone was performed with 0.001% alizarin red solution (Merck Life Science UK Limited) and the tissue further cleared in a 1% potassium hydroxide/20% glycerol solution, increasing to 100% glycerol. Skeletons were examined and images captured using a Leica MZ6 stereomicroscope with a Leica DFC295 camera and the Leica Application Suite software, version 3 (Leica Microsystems UK Ltd, Milton Keynes, UK).

### Single cell mRNA sequencing

The caudal pharyngeal arch region was dissected from four E9.5 embryos and the tissue dissociated to single cells using Accumax (ThermoFisher Scientific) by incubating at 37 °C for 30 min. The reaction was stopped by the addition of 10% fetal calf serum (FCS), and cells were washed in PBS and resuspended in 10% FCS. Fluorescence-activated cell sorting was performed on a Becton Dickinson FACS Aria II using a 100 μm nozzle and a sheath pressure of 20 psi. Single cells were gated using FSC-A versus SSC-A followed by FSC-A versus FSC-H and FSC-A versus SSC-W to remove any doublets. Live single cells were gated using propidium iodide versus FSC-A, and this population was sorted into collection tubes. Single cells were loaded onto the C1 Single-Cell mRNA-Seq HT IFC [10–17 µm] (Fluidigm, San Francisco, CA, USA). Cell lysis, reverse transcription and cDNA amplification were performed using the SMART-Seq v4 Ultra Low Input RNA Kit for the Fluidigm C1 System (Takara Bio USA, Inc, Mountain View, CA, USA). Libraries were pooled and sequenced (2 × 75 bp) on the Ilumina NextSeq 500. Reads were trimmed for quality with Trimmomatic (version 0.33) giving an average of 1,756,297 reads per cell (range 1,057,053–3,185,503). Reads were aligned to the mouse reference genome (GRCm38.p5, version M16) and Ambion spike sequences (1, 4 and 7) using STAR (version 2.4.0j). An average of 1,437,448 reads were uniquely mapped (range 226,060–2,742,686). Reads were quantified using HTSEQ (version 0.6.1) and a count matrix created for analysis (Additional file 5: Table S1).

### Statistical analysis

Fisher’s exact test was used to compare defect frequencies between different genotypes using IBM SPSS Statistics for Windows (Version 25.0; IBM Corp., Armonk, NY, USA). Cell counts were analysed using a one-way ANOVA with Tukey’s multiple comparisons test (Prism 8.01, GraphPad Software, San Diego, CA, USA). Groups were considered significantly different when *p* < 0.05.

## Supplementary Information

**Additional file 1.** Caudal PAA defects in CD1-*Pax9*^*–/–*^ and CD1-*Pax9*^*–/–*^;*Msx1*^*+/–*^ embryos.

**Additional file 2.** Defects in *Pax9*^*–/–*^;*Msx1*^*–/–*^ embryos on a congenic CD1 genetic background.

**Additional file 3.** Apoptosis and cell proliferation are not affected in *Pax9*^*–/–*^ and *Pax9*^*–/–*^;*Msx1*^*+/–*^ mutant embryos on a congenic CD1 background.

**Additional file 4.** Additional Tables S2-4.

**Additional file 5.** Additional Table S1. Count matrix of reads in 86 single cells from the caudal pharyngeal arches of E9.5 mouse embryos.

## Data Availability

The MRI, HREM and μCT imaging datasets analysed during the current study are openly available at https://doi.org/10.25405/data.ncl.c.5431437.v1.

## Abbreviations

AD: Arterial duct
Ao: Aorta
ANOVA: Analysis of variance
cAo: Cervical aortic arch
A-RSA: Aberrant right subclavian artery
BC: Brachiocephalic artery
Bmp4: Bone morphogenetic protein 4
CC: Common carotid artery
CP: Cleft palate
cRSA: Cervical right subclavian artery
dAo: Dorsal aorta
DORV: Double outlet right ventricle
E: Embryonic day
eLC: External left carotid artery
eRC: External right carotid artery
GH: Greater horn (of hyoid)
HREM: High resolution episcopic microscopy
IAA: Interrupted aortic arch
IAA-B: Interrupted aortic arch, type B
IH: Inferior horn (of thyroid cartilage)
iLC: Internal left carotid artery
iRC: Internal right carotid artery
Isl1: Islet 1
IVC: Interventricular communication
LCC: Left common carotid artery
LH: Lesser horn (of hyoid)
LSA: Left subclavian artery
LV: Left ventricle
MRI: Magnetic resonance imaging
Msx1: Muscle segment homeobox 1
RCC: Right common carotid artery
RE-RSA: Retro-esophageal right subclavian artery
RSA: Right subclavian artery
RV: Right ventricle
NCC: Neural crest cell
P: Palate
PA: Pharyngeal arch
PAA: Pharyngeal arch artery
Pax9: Paired box 9
PSC: Primitive subclavian complex
PT: Pulmonary trunk
Runx2: Runt-related transcription factor 2
SHF: Second heart field
SH: Superior horn (of thyroid cartilage)
SMC: Smooth muscle cells
Tbx1: T-box transcription factor 1
Th: Thymus
U: Ulna
VSD: Ventricular septal defect
Wnt1: Wingless-type MMTV integration site family, member 1
