# Supplementary material for: Msx1 haploinsufficiency modifies the Pax9-deficient cardiovascular phenotype
Source: BMC Dev Biol. 2021 Oct 6;21:14. doi: 10.1186/s12861-021-00245-5 (PMC8493722; doi:10.1186/s12861-021-00245-5)
Supplement: Supplementary file 1 — Additional file 1. Caudal PAA defects in CD1-Pax9–/– and CD1-Pax9–/–;Msx1+/– embryos. [file 12861_2021_245_MOESM1_ESM.docx]

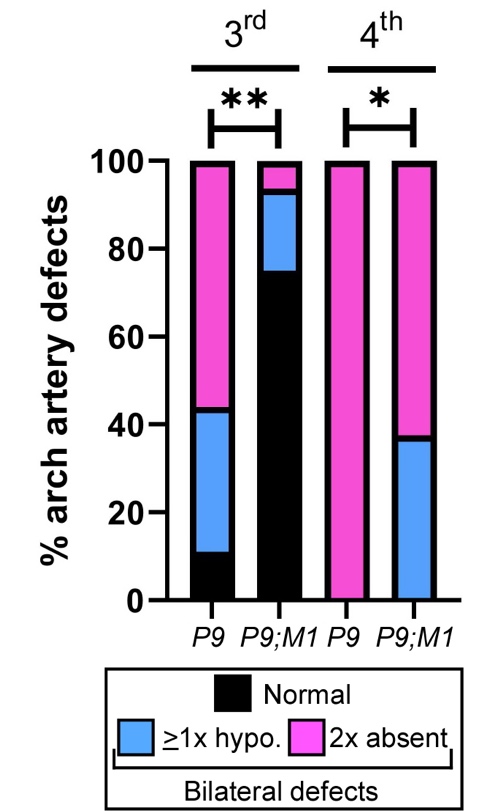


**Additional file 1.** **Caudal** **PAA defects in CD1-*Pax9^–/–^* and *CD1-Pax9^–/–^;Msx1^+/–^* embryos**

The phenotype of the 3^rd^ and 4^th^ PAAs were recorded in E10.5 embryos following intracardiac ink injection. Data is reproduced from Table 3 and the incidence expressed as a percentage. The PAAs are defined as normal when both left and right PAAs are patent to ink and of normal size, hypoplastic (hypo.) if thinner than normal, and absent when not patent to ink. There is a significant increase in normal 3^rd^ and hypoplastic 4^th^ PAAs in *CD1-Pax9^–/–^;Msx1^+/–^* embryos (*P9;M1*) compared to CD1-*Pax9^–/–^* embryos (*P9*). Fisher’s exact test for associations, **p<0.005, **p<0.01.
