# Supplementary material for: Msx1 haploinsufficiency modifies the Pax9-deficient cardiovascular phenotype
Source: BMC Dev Biol. 2021 Oct 6;21:14. doi: 10.1186/s12861-021-00245-5 (PMC8493722; doi:10.1186/s12861-021-00245-5)
Supplement: Supplementary file 2 — Additional file 2. Defects in Pax9–/–;Msx1–/– embryos on a congenic CD1 genetic background. [file 12861_2021_245_MOESM2_ESM.docx]

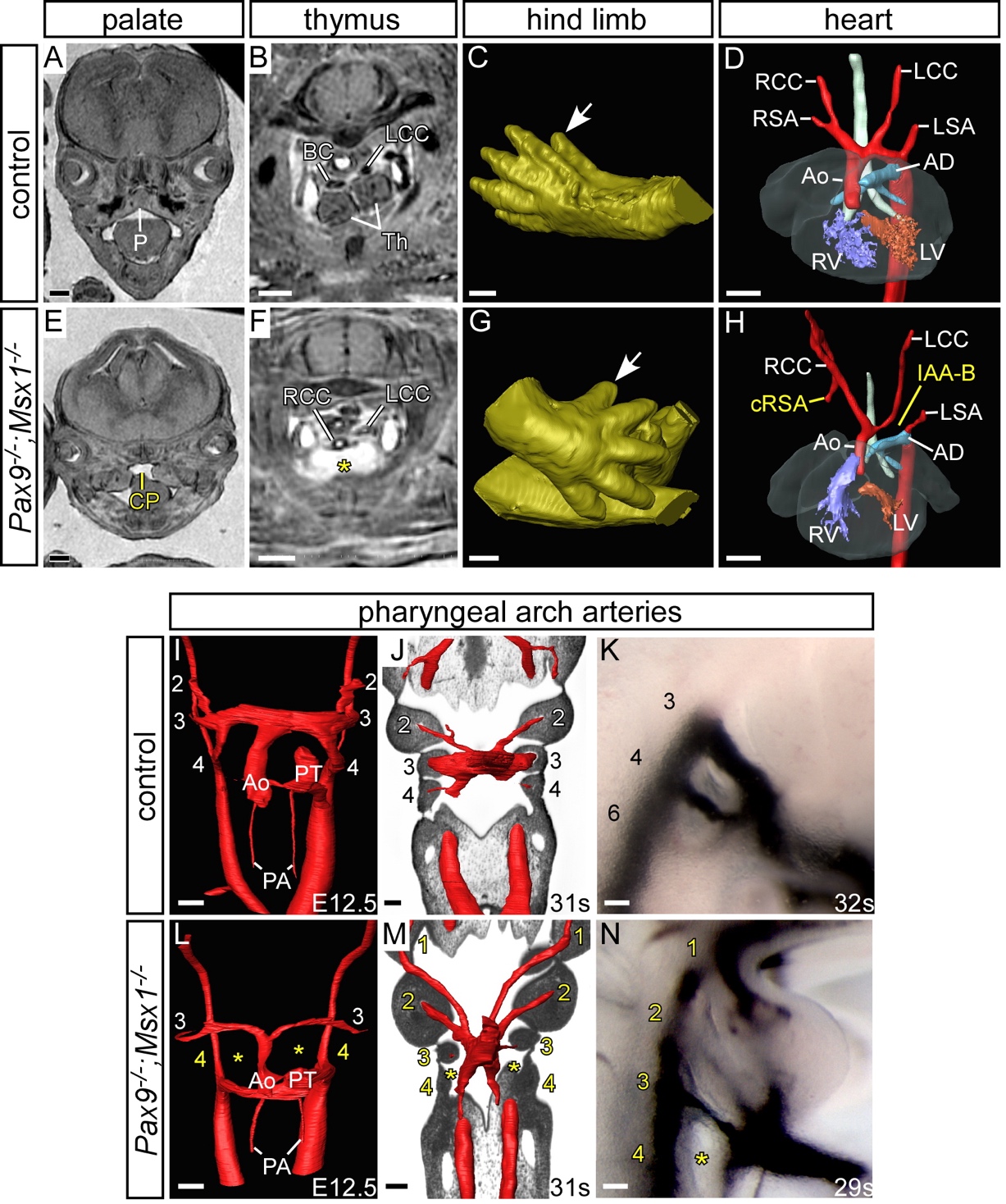


**Additional file 2.** **Defects in *Pax9^–/–^;Msx1^–/–^* embryos on a congenic CD1 genetic background.**

Embryos at E15.5 were imaged by MRI (A-H), at E12.5 (I, L) and E10.5 (J, M) by HREM, and at E10.5 following intracardiac ink injection (K, N). Panels A-D are taken from Figure 1 for comparison. (A-D) Control embryos have a normal palate (A), thymus (B), hind limb digit (white arrow; C), heart and arch arteries (D). (E-H) *Pax9^–/–^;Msx1^–/–^* embryos display cleft palate (CP; E), absent thymus (asterisk; F) but do not have pre-axial digit duplication (white arrow; G) as seen in *Pax9^–/–^* and *Pax9^–/–^;Msx1^+/–^* embryos (see Figs 1H, 5D and 6B,C). (H) Some *Pax9^–/–^;Msx1^–/–^* embryos have cardiovascular defects such as cervical origin of the right subclavian artery (cRSA) and interrupted aortic arch type B (IAA-B). (I-K) Control embryos showing normal pharyngeal arch artery (PAA) development. (I) Embryo at E12.5 with a septated outflow tract and symmetric 3^rd^ and 4^th^ PAAs. (J) Embryo at E10.0 with symmetric 2^nd^ and 3^rd^ PAAs. The 4^th^ PAA is forming. (K) Embryo at E10.0 with symmetrical 3^rd^, 4^th^ and 6^th^ PAAs patent to ink. (L-N) *Pax9^–/–^;Msx1^–/–^* embryos have aberrant PAA morphogenesis. (L) E12.5 embryo with hypoplastic 3^rd^ and absent 4^th^ PAAs. The outflow tract is not fully septated. (M, N) E10.0 embryos with aberrantly persisting 1^st^ and 2^nd^, hypoplastic 3^rd^ and absent 4^th^ PAAs.

Abbreviations: AD, arterial duct; Ao, aorta: LCC/RCC, left/right common carotid artery; LSA/RSA, left/right subclavian artery; LV/RV, left/right ventricle; P, palate; PA, pulmonary artery; PT, pulmonary trunk; Th, thymus. Scale bars: 500µm in A-H, 100µm in I-N.
