# Supplementary material for: Msx1 haploinsufficiency modifies the Pax9-deficient cardiovascular phenotype
Source: BMC Dev Biol. 2021 Oct 6;21:14. doi: 10.1186/s12861-021-00245-5 (PMC8493722; doi:10.1186/s12861-021-00245-5)
Supplement: Supplementary file 3 — Additional file 3. Apoptosis and cell proliferation are not affected in Pax9–/– and Pax9–/–;Msx1+/– mutant embryos on a congenic CD1 background. [file 12861_2021_245_MOESM3_ESM.docx]

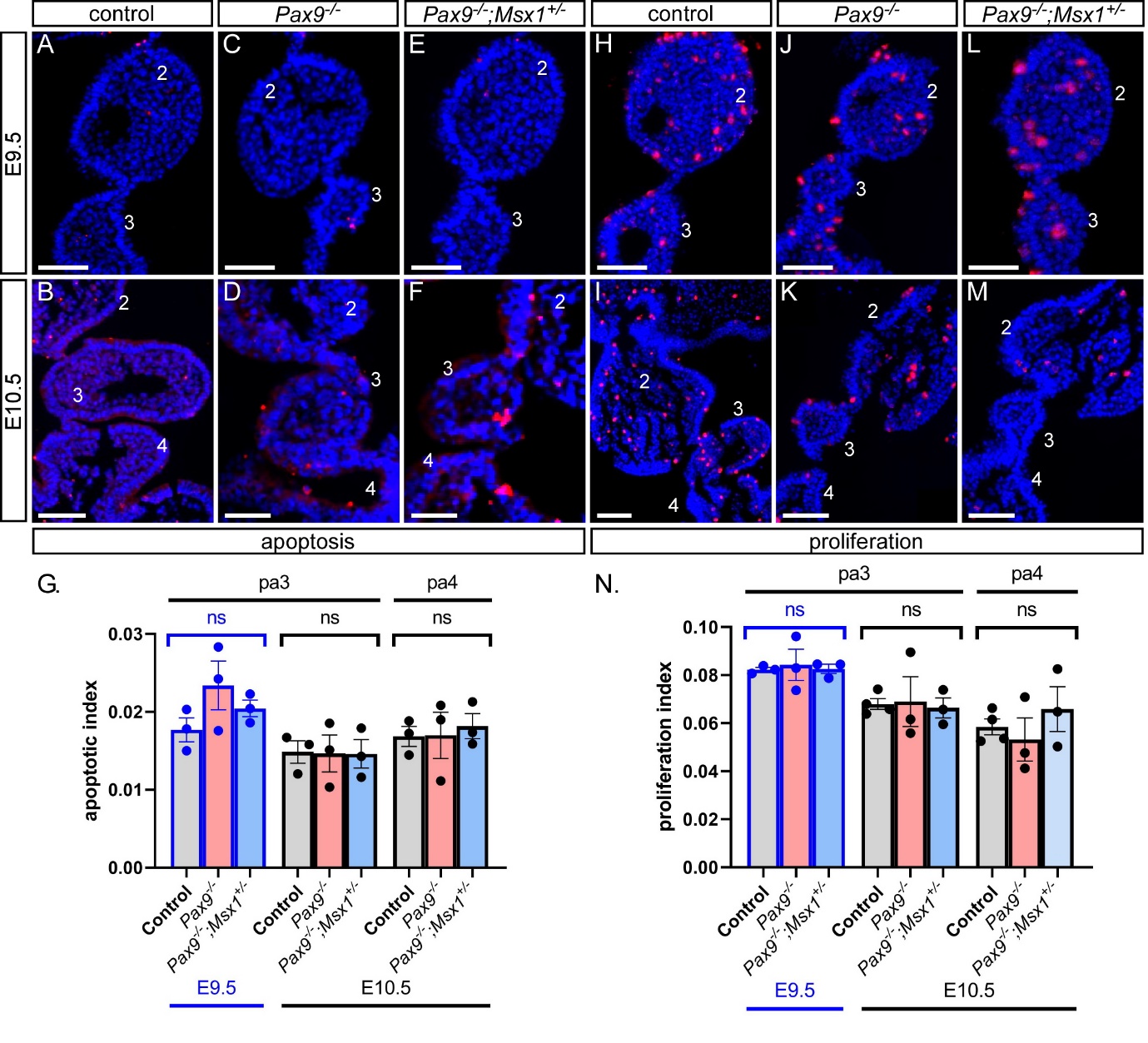


**Additional file 3. Apoptosis and cell proliferation are not affected in *Pax9^–/–^* and *Pax9^–/–^;Msx1^+/–^* mutant embryos on a congenic CD1 background.**

(A-F) Embryo sections were immunostained using an anti-caspase-3 antibody to detect apoptosis in the caudal pharyngeal arches of control (A, B), *Pax9^–/–^* (C, D) and *Pax9^–/–^;Msx1^+/–^* (E, F) embryos at E9.5 and E10.5. (G) No significant difference in the rate of apoptosis was found between control, CD1-*Pax9^–/–^* and CD1-*Pax9^–/–^;Msx1^+/–^* embryos at E9.5 and E10.5 (n=3 per genotype and stage). (H-M) Embryo sections were immunostained using an anti-phospho-histone H3 antibody to detect proliferation in the caudal pharyngeal arches of control (H, I), *Pax9^–/–^* (J, K) and *Pax9^–/–^;Msx1^+/–^* (L, M) embryos at E9.5 and E10.5. (N) No significant difference in the rate of proliferation was found between control, CD1-*Pax9^–/–^* and CD1-*Pax9^–/–^;Msx1^+/–^* embryos at E9.5 and E10.5 (n=3 per genotype and stage). Each pharyngeal arch was defined by the boundary formed by the pharyngeal pouch and cleft. The apoptotic and proliferative indices were calculated by counting the number of positively stained cells divided by the total number of DAPI stained cells within each pharyngeal arch section, and the mean of these counts calculated. One-way ANOVA with Tukey’s multiple comparisons test. ns, not significant; pa, pharyngeal arch. Scale bars: 50μm.
