# Supplementary material for: Msx1 haploinsufficiency modifies the Pax9-deficient cardiovascular phenotype
Source: BMC Dev Biol. 2021 Oct 6;21:14. doi: 10.1186/s12861-021-00245-5 (PMC8493722; doi:10.1186/s12861-021-00245-5)
Supplement: Supplementary file 4 — Additional file 4. Additional Tables S2-4. [file 12861_2021_245_MOESM4_ESM.docx]

**Additional Tables**

**Additional Table S1**. Count matrix of reads in 86 single cells from the caudal pharyngeal arches of E9.5 mouse embryos (separate Excel file).

**Additional Table S2**. Developmental defects in *Msx1^–/–^* and *Pax9^+/–^:Msx1^–/–^* mutant mice

|  |  |  | **Defects** | | | |
| --- | --- | --- | --- | --- | --- | --- |
| **Genotype** | **Stage** | **n** | **Palate** | **Thymus** | **Limb** | **Cardio** |
| *Msx1^–/–^* | Neonate | 6 | 7  (100%) | 0 | 0 | 0 |
|  | E15.5 | 1 |  |  |  |  |
| *Pax9^+/–^;Msx1^–/–^* | Neonate | 8 | 14  (100%) | 0 | 0 | 0 |
|  | E15.5 | 6 |  |  |  |  |
|  | E10.5 | 7 | - | - | - | 0 |

Defects were assessed in neonates by direct observation, E15.5 embryos by μCT and MRI analysis, and the pharyngeal arch arteries in E10.5 embryos by intra-cardiac ink injection. Abbreviation: Cardio, cardiovascular.

**Additional Table S3**. PCR primers for genotyping

| **Mouse line** | **Primer name** | **Primer sequence** | **Product (bp)** |
| --- | --- | --- | --- |
| *Pax9* | P9-gen2-F1 | ACTCACCGGCCTGCACCAATTAC | 196 (wild-type) 450 (mutant) |
|  | P9-gen2-R1 | TTGTTCTCACTGAGCCGGCCTGT |  |
|  | P9-gen2-R2 | GGATGTGCTGCAAGGCGATTAAG |  |
| *Pax9^Flox^* | P9-lox1-F | AGCGGAGACAAGGATGAAACCAC | 305 (wild-type) 351 (mutant) |
|  | P9-lox1-R | AGAGGAATCCCGATGTTCACCAG |  |
| *Msx1* | M1-F1b | CCTACGCAAGCACAAGACCAAC | 202 (wild-type) 410 (mutant) |
|  | M1-R1b | CTCCTGCAGTCTCTTGGCCTTA |  |
|  | M1-R2b | GGCCACACGCGTCACCTTAATA |  |
| *Isl1Cre* | S1X-A | GCATAACCAGTGAAACAGCATTGCTG | 280 |
|  | S1X-B | GGACATGTTCAGGGATCGCCAGGCG |  |

**Additional Table S4.** Antibodies used for immunostaining

| **Target** | **Catalogue number** | **Species and type** | **Supplier** | **Dilution** |
| --- | --- | --- | --- | --- |
| *Primary antibodies* | | | | |
| AP-2α | 3B5 | Rat monoclonal | Santa Cruz | 1:50 |
| aSMA | a2547 | Mouse monoclonal | Sigma | 1:500 |
| Cleaved caspase-3 | 9661 | Rabbit polyclonal | Cell Signalling | 1:100 |
| ERG | ab92513 | Rabbit monoclonal | Abcam | 1:200 |
| Phospho-histone H3 | 06-570 | Rabbit polyclonal | Millipore | 1:300 |
| *Secondary antibodies* | | | | |
| Donkey anti-mouse IgG Alexa Fluor 594 | A-21203 | - | Thermo Fisher Scientific | 1:200 |
| Donkey anti-rabbit IgG Alexa Fluor 594 | A-21207 | - |  |  |
| Donkey anti-rat IgG Alexa Fluor 594 | A-21209 | - |  |  |
| Donkey anti-rabbit IgG Alexa Fluor 488 | A-21206 | - |  |  |
| *Nuclear stain* | | | | |
| DAPI | H-1200 | - | Vector Laboratories | - |
